# Synthesis, characterization and in vitro release kinetics of methotrexate from oxidized fenugreek gum/chitosan-catechin bionanocomposite hydrogel matrix

**DOI:** 10.1186/s11671-026-04698-0

**Published:** 2026-06-24

**Authors:** Ganesh Kumar, Nisha Sharma, Younis Ahmad Hajam

**Affiliations:** 1 Department of Physical Sciences, Sant Baba Bhag Singh University, Jalandhar, Punjab 144030 India; 2 Department of Life Sciences, Sant Baba Bhag Singh University, Jalandhar, Punjab 144030 India

**Keywords:** Catechin, Bio nanocomposite, Fenugreek gum, Natural antioxidant, Biocompatibility

## Abstract

Functionalized polysaccharide-based nanocomposite hydrogels have been utilized as drug delivery devices to achieve controlled release of drug moieties during the treatment of cancer. To achieve optimum drug release behaviour, drug carrier should have tunable swelling profile, biodegradability, non-toxic nature, flexibility like soft tissues, and biocompatibility. In present study, catechin-embedded oxidized fenugreek gum/chitosan bio nanocomposite matrix had been synthesized. For synthesis of catechin embedded hydrogel, catechin (~ 2.30%) was extracted from *Acacia catechu* heartwood and confirmed by LC-MS/HPLC along with other phyto-chemicals.structural modifications achieved in synthesized gel matrix had been analyzed using FESEM, FTIR, XRD, TEM and TGA analysis. Antioxidant property of gel matrix viz.: OFG-*cl*-CH (OFC), catechin, and OFG-*cl*-CH/catechin bio nanocomposite (OFCC) matrix was examined by DPPH assay. In vitro drug release behaviour of methotrexate (MTX) an anticancer drug was evaluated from functionalized gels. Incorporation of catechin onto oxidized fenugreek gum–chitosan hydrogel (OFC) via Schiff base cross-linking achieved 53.23% encapsulation, improved crystallinity, stability, and morphology. OFCC hydrogel had depicted pH-sensitive swelling profile (max 115.20% at pH 2.2) and sustained MTX release in acidic media (75.19% in Simulated Gastrc fluid (SGF)upto 24 h) following Korsmeyer–Peppas release kinetics. All hydrogels (FG, OFG, OFC, OFCC) showed excellent biocompatibility, with non-thrombogenic behaviour, low clot weights, and hemolysis indices < 5%, confirming their safety for biomedical use. Catechin exhibited strong antioxidant activity (77%), which was enhanced upon incorporation into the Schiff base matrix, with a slight dose-dependent improvement. Biodegradability tests revealed that oxidation, cross-linking, and catechin incorporation progressively reduced degradation, with OFCC showing maximum stability. Overall, OFCC hydrogel improves catechin stability, enhances matrix crystallinity, thermal and morphological properties, thus emerged as a biocompatible, antioxidant, and durable hydrogel with strong potential for sustained release, making it a promising system for pH-responsive anticancer drug delivery in gastric conditions.

**Supplementary Information:**

The online version contains supplementary material available at 10.1186/s11671-026-04698-0.

## Introduction

Cancer is the leading cause of global mortality, with chemotherapy, radiation, and surgical amputation being widely used treatments. These methods, involving high-intensity radiation and medications, can cause side effects and decrease patient compliance [1]. Cancer drugs adopt different mode of action such as alkylation, oxidative degradation, antitumor antibiotics, anti-metabolites, dissolution of mitotic assembly structure and topoisomerase inhibitor etc. [2].Cancer therapeutics are hydrophobic in nature such as paclitaxel, docetaxel, camptothecin, doxorubicin, curcumin etc., making them difficult to deliver in required concentration and maintaining efficacy in systemic circulation [3]. To address such issues, sophisticated drug delivery systems are desirable to maintain concentration, minimize adverse effects, and incorporate anticancer medicine in suitable carriers. MTX is a highly powerful drug for treatment of inflammatory arthritis and other solid tumors. Direct administration of the drug by conventional drug delivery system leads to decrease in the therapeutic efficiency due to hydrophobic nature of MTX [4]. Suitable drug delivery device provides safer solution to improve half-life and enhance efficiency of MTX and provide sustained release at the particular site [5].

Hydrogels being advanced materials have been extensively exploited as suitable drug carriers due to their tunable swelling profile, biodegradability, non-toxic nature, flexibility similar to soft tissues, variable chemical structures, high water content and soft consistency thus reflecting biocompatibility [6]. Despite these excellent properties, polysaccharide-based hydrogels encounter some limitations such as uncontrolled swelling profile, poor mechanical and tensile properties.Different functionalization procedures have been employed in literature to increase stimulus responsiveness and texture behaviour of hydrogels. Reinforcement through the incorporation/functionalization of nanoparticles (organic/metallic/inorganic/clay/carbon based) results in the production of nanocomposite hydrogels which possess improvedcharacteristics some of which become suitable for biomedical use. In literature different nanocomponents such as inorganic [7], organic, metal [8], metal oxides [9], and natural extracts which have been employed as reinforcing agents in nanocomposite hydrogel fabrication.

Nanocomposite hydrogel have been exploited in different sectors such as biomedical applications, including regenerative medicine, bio-imaging, tissue/wound management, medication delivery systems, energy storage, food packaging materials, and bioelectronics [10].In drug delivery systems, nanoparticles assist in maintenance of structural integrity of the gel matrix, improve stability of drugs, diminish the burst drug release effect, and provide a slower, safer and prolonged release [11]. Such functionalized nanocomposite hydrogels also improvemechanical strength and biocompatibility behaviour as well as manage drug toxicity [12]. In current scenario, with respect to environmental concerns, green methods and ecofriendly materials are preferred choice for fabrication of drug delivery system. Contrary to conventional synthesis procedure such as graft copolymerization, self-cross-linking approach is more acceptable for hydrogel synthesis. Oxidative process of hydrogel fabrication is exploited recently by various researchers since this route results in selective oxidation of polymeric backbone. Reactive sites (preferrably carbonyl groups) generated via oxidation of polysaccharide undergo self-cross-linking with compatible functional groups such as amino groups of the second component of the hydrogel system, avoiding the use of harsh chemical cross-linkers or initiator systems, as required in conventional method of hydrogel synthesis. In literature various plant based gums have been functionalized via oxidative route. It has been reported that oxidised sodium alginate was oxidized with sodium periodate [13].Oxidised gum arabic has better porosity and mechanical qualities than native gum arabic [14]. Oxidised dialdehyde inulin-adipic acid dihydrazide matrix has good bioavailability and pharmacokinetic features for 5-fluorouracil (5-FU) release, as well as lower toxicity [15]. Sodium alginate-chitosan (Na Alg-CS) polymeric complex loaded with methotrexate was created via ionotropic gelation resulting in biocompatible and biodegradable matrix which had demonstrated sustained drug release over time [16]. In another study, sodium alginate-sodium carboxymethyl cellulose composite hydrogels have also been used as biocompatible drug carriers for the controlled release of MTX [17].

Polyphenols like catechin has several therapeutic effects, including antioxidant, anticancer, and anti-inflammatory activity [18]. Catechin scavenges free radicals and generates singlet oxygen, hydroxyl radicals, and superoxide free radicals [19].Catechin inhibits the lipid oxidation of red meat, poultry, and fish, making them an ideal natural food preservative in the food industry [20]. Despite their biological properties, Catechin has low aqueous solubility which restricts their bioavailability and hence clinical efficiency [21]. Polysaccharide based gel serve as suitable encapsulating matrix for polyphenols resulting in formation of hybrid composite gel with integrated properties of all components. Encapsulation of catechin in gel matrix is a preferable choice for catechin usage in the pharmaceutical sector.

**Fig. 1:**
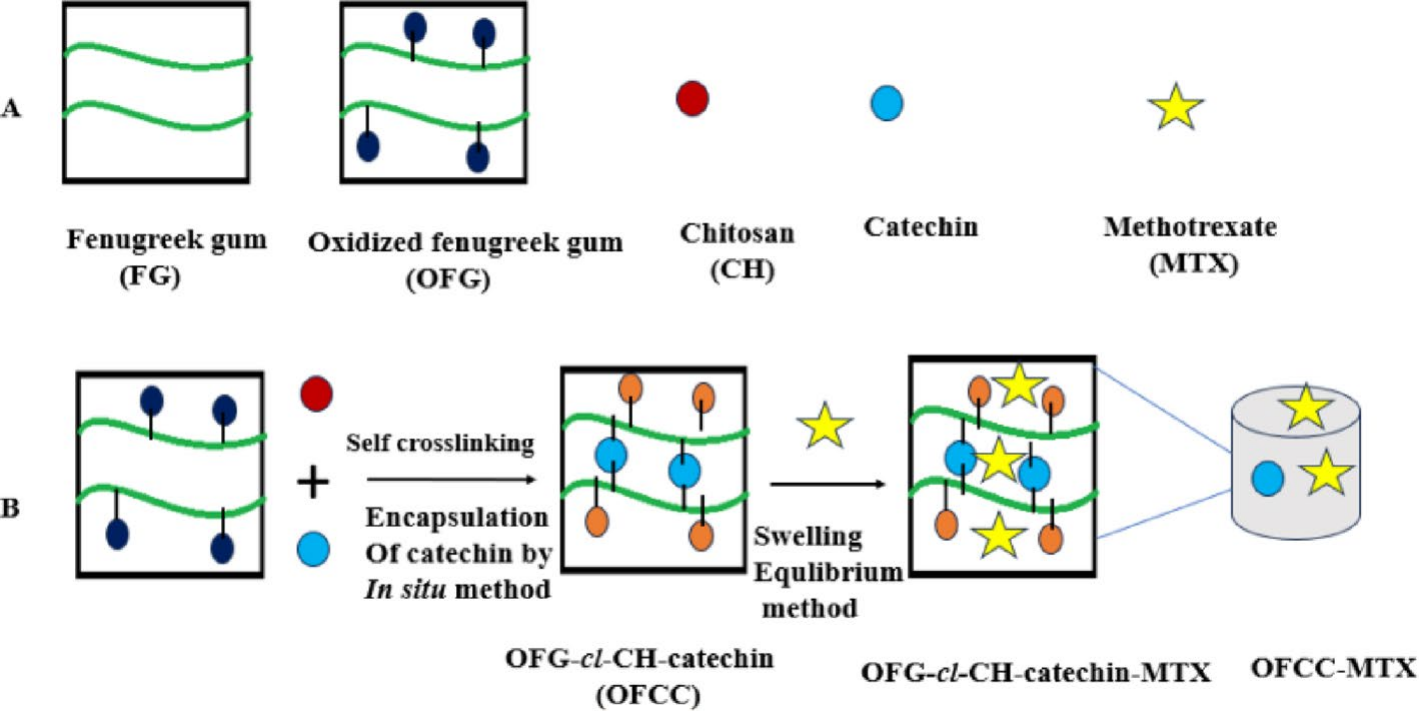
Schematic illustration of the** A** components and** B** formation of OFCC and OFCC-MTX

Fenugreek gum (FG) is a glactomannan seed gum, which is similar to guar gum, tara gum, and locust bean gum [22]. It is composed of equimolar 1:1 ration of linear β (1–4) mannose and α (1–6) galactose, connected via (1–6) glycosidic bond (Fig. 1). FG is highly soluble gum among all galactomannans, with high viscosity and swelling ability, thus form highly viscous swelling gel in aqueous medium due to strong hydrogen bonding [23]. Therapeutically, FG is utilized as a primary home medicine because of its antidiabetic, antibacterial, antioxidant and anticarcinogenic activities.

Chitosan (CH) is a naturally occurring cationic natural polymer and extensively used in biomedical applications due to its biocompatible, nontoxic and antimicrobial behaviour. Due to the presence of a free amino group on the CH backbone, it has been used as a cross-linking centre in the manufacture of hydrogel with periodate oxidised polysaccharide backbones. Periodate-oxidized starch and chitosan resulted in self-cross-linking polymeric gel [24].In another study, thiolated chitosan and oxidised dextran gum had resulted in bi-functional in situ self-cross-linked polymeric hydrogel network without the need of chemical cross-linkers [25].A dextran dialdehyde–chitosan adhesive biomaterial was developed through a Schiff base reaction as well. This matrix served as efficient haemostatic matrix, thus encourages tissues bonding well with minimal cytotoxicity. In addition to its tissue sealing capabilities, this matrix could be exploited as a biopolymeric vehicle for drug delivery [26]. Similarly, oxidized konjac glucomannan cross-linked chitosan based self-crosslinked Schiff’s base hydrogel worked as biocompatible drug carriers for in vitro release of ofloxacin [27].

Keeping in view the therapeutic significance of fenugreek gum, chitosan, and catechin, current study focuses on the synthesis of a novel bio nanocomposite matrix composed of fenugreek gum/chitosan embedded with freshly extracted catechin from *Acacia catechu* via a self-cross-linking approach using the Schiff’sbase reaction. Newly fabricated matrix was utilized for in vitro release of methotrexate (MTX), an anticancer drug.The schematic illustration of the components and formation of OFCC and OFCC-MTX matrix have been shown in Fig. 1. Drug release kinetics and dynamics have been investigated in a different release media. Novelty of the work is based on the fact that both materials and method of gel synthesis was achieved without using a chemical cross-linker. To the best of our knowledge, there is no published report on the development of a fenugreek gum-chitosan-catechin bio nanocomposite gel matrix. The biocompatible/hydrolytic degradation and antioxidant behaviour of the synthesized nanocomposite was also evaluated in the present study.

## Materials and methods

*Acacia catechu* (Khair wood) was collected from Una, and Himachal Pradesh, gifted by Khanna Katha Udyog, Una, and Himachal Pradesh and identified Dr. Vikrant Jaryan (Associate Professor), Department of Agriculture, Sant Baba Bhag Singh University, Khiala, Jalandhar. The species is not protected and not listed as endangered. Therefore, no specific permissions or licenses were required for its collection. All procedures complied with relevant institutional, national, and international guidelines and legislation. Fenugreek gum (FG) was obtained from Chemtotal Labs Pvt.Ltd., Rajasthan India. Ethylene glycol and ethyl acetate were purchased from CDH Pvt.Ltd., New Delhi, India. Sodium periodate was procured from Nice Chemicals Pvt.Ltd., Kochi, India. Ethanol, Calcium Chloride (CaCl_2_) and 37% Formaldehyde were obtained from Fine Chemicals Private Limited, Mumbai, India. Chitosan (CH) (Deacetylated > 95%) was purchased from Benzochem industries Pvt.Ltd. Maharashtra. Sodium chloride, Sodium hydroxide and Potassium chloride were procured from Merck chemicals. Potassium dihydrogen orthophosphate (KH_2_PO_4_), and Hydrochloric acid (HCl) were purchased from Rankem Pvt. Ltd., New Delhi; India. Phosphate buffer saline (PBS, pH 7.4) was purchased from Ayonex Labs Pvt.Ltd., Zirakpur; Punjab. Pancreatin was purchased from Loba Chemie Pvt. Ltd. and pepsin (SD-Fine chemical Ltd.Mumbai-India) was used as received. The model drug methotrexate (MTX) (Ipca Laboratories Limited, Shankarpur; Uttrakhand, India) was purchased from a local pharmacy. Catechin reference standard was purchased from Sigma-Aldrich. All chemicals were of analytical grade and used as such without any further purification. Double distilled water (DDW) was used in the preparation of all solutions.

Buffer solutions of pH (2.2, 6.8 and 7.4) and 0.9% NaCl solutions were prepared as reported in the Pharmacopoeia of India [28]. Simulated gastric fluid of pH 1.2 containing pepsin and simulated intestinal fluid of pH 6.8 containing pancreatin were prepared according to the procedure reported in United States Pharmacopeia and National Formulary [28].

### Preparation of catechin

Catechin was extracted from *Acacia catechu* via method as reported previously with minor modifications [29, 30]. Briefly, fine chips of heartwood of *Acacia catechu* (50 g) were extracted in 150 ml DDW using Soxhlet apparatus. Dark brown solution of extract was subsequently filtered through a muslin cloth. Resultant liquid extract was concentrated one-fourth volume and stored 2–3 °C for four days. Crystallized slurry thus obtained was filtered using Buchner funnel with the help of vacuum pump. Resultant crystalline material was submerged in DDW for overnight, followed by heating up to 70 °C with activated charcoal to remove any colored impurities, filtered using whatman filter paper No.41 and refrigerated at 2–3 °C overnight (for12h). Crystalline extract was dried & stored at room temperature. Crude product of *Acacia catechu* extract (4 g) was mixed with 12 ml of DDW and grinded with pestle mortar followed by soaking in ethyl acetate (50 ml) for two days followed by filtration through whatman filter paper no.41. The solvent was evaporated by distillation to produce the ethyl acetate extract and concentrated subsequently through simple distillation. Thick liquid (14 ml) thus obtained was refrigerated at 2–3 °C overnight (undisturbed for12h) before being filtered and washed three times with cold DDW. The precipitates of catechin were air dried and kept for later usage in a moisture-free environment (Supplementary Fig. S1).

### Synthesis of OFG-*cl-*CH/catechin(OFCC) hydrogel

In the present study, fenugreek gum/chitosan hydrogel matrix (OFC)) was synthesized via Schiff’s base formation between oxidised fenugreek gum and chitosan, as described in our previous paper [31]. Briefly, 2% FG (100 ml, w/v) was oxidized using 0.05 M sodium periodate (25 ml) under continuous stirring condition using magnetic stirrer (1MLH, Remi equipment’s Pvt. Ltd., India) for 24 h at room temperature under dark conditions. After stipulated time, reaction was terminated using ethylene glycol (5 ml), followed by precipitation using excess of ethanol (approx.500 ml). Resultant OFG was filtered & washed with DDW to remove unreacted oxidant followed by air drying at .room temperature and further preserved in vacuum desiccators.

In second step, OFG (0.5 g) was dispersed in DDW (25 ml) under stirring condition at 40 °C for 2 h followed by addition of solution of chitosan (1 g in 25 ml dispersed in 2% acetic acid). The reaction mixture stirred at 40 °C for 5 h resulted into homogenous mixture which was transferred to a glass petri dish and refrigerated for 5 min. Afterward, within 30–35 min, self-crosslinking among reactive functional groups of both polymeric constituents was achieved at room temperature resulting into OFG-*cl*-CH matrix (OFC). Formed gel matrix was washed with DDW, dried under room temperature and stored in moisture free conditions for further use (Supplementary Figure S2(a-b).

**Fig. 2:**
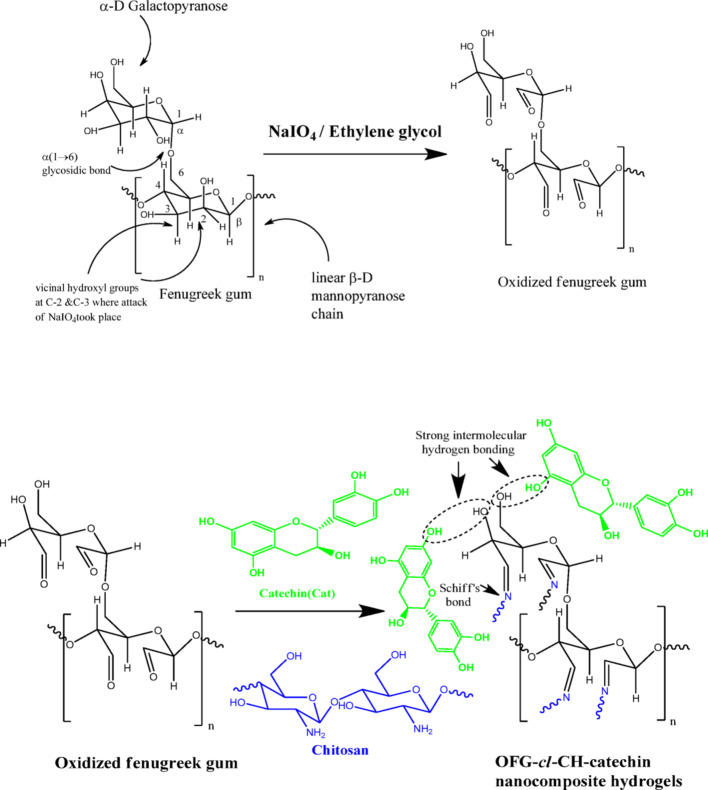
Plausible mechanism of synthesis of OFG*-cl*-CH-catechin nanocomposite hydrogel by *insitu* method

OFG*-cl*-CH/catechin bionanocomposite gel matrix (OFCC) had been fabricated via in situ cross-linking method using two different concentrations of catechin viz.: 2500 µg/ml and 5000 µg/ml [32]. Briefly, OFG (0.5 g) was dispersed in DDW (25 ml) under continuously stirring at 40 °C for 2 h. Similarly, chitosan dispersion (1 g in 25 ml dispersed in 2% acetic acid) was prepared under continuous stirring condition for 2 h at 40 °C. Freshly extracted catechin powder (25 mg and 50 mg) were dispersed in 10 ml of ethanol each and preserved in a dark at room temperature. Afterward, dispersions of OFG, CH, and catechin(2500 µg/ml & 5000 µg/ml) were mixed separately under continuous stirring at 40°Cfor 5 h to achieve a homogeneous solutions [33]. Both reaction mixtures then transferred to a glass petri dish, refrigerated for 5 min. Within 30–35 min, two cross-linked bionanocomposite gel matrix embedded with 2500 µg/ml and 5000 µg/ml catechin respectively were formed via self-cross-linking technique. Resulted gel networks were dried at room temperature, stored in a zero-humidity condition and designated as OFG*-cl*-CH-catechin bio-nanocomposite hydrogel (OFCC) (Fig. 2, Supplementary Fig. S2(d), S3).

In addition to in situ method, OFCC gel was also fabricated through swelling equilibrium method [34]. In this method dried OFC (300 mg) was immersed in2500µg/ml catechin solution (50 ml), allowed to swell for 24 h at room temperature. During swelling, catechin molecules got absorbed and entrapped in the gel networks and resulting in colour change of OFC gel from light yellow to light brownish color. Swollen hydrogel was dried, stored under dry condition for further study. Encapsulation efficiency (EE%) of catechin loaded hydrogel prepared via swelling equilibrium method was analyzed spectrophotometrically usingdouble beam UV-Visible Spectrophotometer(Elico SL-164). Cconcentration of unentrapped catechin was estimated from a standard calibration curve by measuring the absorbance at λ max=280 nm [35]. EE % was calculated using Eq. (1).

1 $$ EE\% = \frac{{{\text{Total amount of catechin in OFG}} - {\mathrm{c}}l - {\mathrm{CH}}/{\text{catechin hydrogel}}}}{{{\text{Amount of catechin taken for loading}}}} \times 100 $$

The composition of OFC, OFCC, and drug loaded OFC-MTX and OFCC-MTX gel matrix have been given in supplementary Table S1(a).

### Characterization

Structural, functional group characterizations of FG, OFG, CH and OFC have already reported in our previous published article [30]. In present work, drug loaded OFG-*cl*-CH-MTX (OFC-MTX) and OFG-*cl*-CH-Catechin-MTX (OFCC-MTX) were characterized by Fourier-transform infrared spectroscopy (FTIR), powdered X-ray diffraction method (XRD), Field emission scanning electron microscopy (FESEM), Transmission electron microscopy (TEM) and Thermo gravimetric analysis (TGA).FTIR spectra were recorded using Perkin Elmer FTIR Spectrometer of version 10.6.1 using KBr pallet method in the range of 4000–400 cm^− 1^ with a resolution of 2 cm^-1^.Bruker X-ray diffractometer employing Cu Kα radiation was used to study powdered X-ray diffraction patternin the scan range from 10–60° (2θ) at ambient condition. Perkin Elmer Thermo gravimetric analyzer was used to study the change in the thermal properties of the hydrogel after the incorporation of catechin nanoparticles.TGA had been conducted in temperature gap ranged from room temperature to 700 °C at a heating rate of 10 °C/min under inert atmosphere (nitrogen gas).The surface morphology of nanocomposite hydrogel was analysed using FESEM (JEOL, Japan) which was operated at accelerating voltage of (0.1–30KV) and crystallinesize of incorporated catechin was confirmed by TEM (Hitachi, H-7500) with 40–120 operating voltage.

#### Preliminary phytochemical analysis of catechin

The preliminary phytochemical tests were performed for the identifications of the different secondary metabolites in extracted catechin according to standard methods as described in literature [36, 37].

#### Characterization of catechin by LC/MS analysis

In present work, catechin extracted from *Acacia catechu* heartwood was analyzed by mass spectrometry analysis using positive ionization mode with m/z in the range of 100–2000 by LC-MS (Water’s SYNAPT-XSHDMS, UK) at SAIF, Panjab University Chandigarh, according to the chromatographic method [38]. In order to perform LC-MS analysis, 10 mg of the extract sample was diluted in about 100 ml of diluents (0.1% formic acid water and Methanol, 3:7) and filtered through a 0.45 μm filter. Each investigation uses a 5 µl injection volume. An extract aliquot was put onto the LC-MS column. The chromatographic separation’s mobile phase is made up of solvents A and B, which contain 0.1% formic acid in LC-MS grade water and 0.1% formic acid in acetonitrile, respectively. The mass spectrometer was coupled with HPLC (Waters 2795) with quaternary pumping set up for 0.2 ml/min of flow.10% B was used to neutralize the column for 10 min prior to the subsequent injection procedure. Eluent was monitored using ESI-MS. The sample was scanned from m/z 100 to 2000.

#### Characterization of catechin by high-performance liquid chromatography (HPLC) analysis

Catechin extracted was also analyzed through HPLC according to procedure reported in the literature with some modifications [39]. The study used HPLC (2489 system: Water’s USA) equipped with a binary pump, photodiode array detector, auto-sampler, and inline degasser for chromatographic separation. Standard and sample were dissolved in methanol, filtered, and solvents used for elution were filtered before injection. The sample and standard were eluted using a gradient solvent program for 55 min at room temperature. The solvents were A: B, (2% acetic acid (v/v) in water and 1% acetic acid (v/v) in water: acetonitrile (1:1, v/v) constitute the solvent A and B, respectively) with varying concentrations for different durations. The elution gradient was varied from 0 to 55 min, with the final concentration being A: B (0: 100).Phenolic compounds were detected at 280 nm spectrophotometrically. The peak of each phenolic compound was identified with the authentic standards by comparing the retention time (RT) and their quantification was carried out using standard curves and peak area.

### Swelling kinetics of functionalized OFG*-cl-*CH (OFC) gel matrix

The swelling behaviour of catechin functionalized OFC gel matrix (in situ/swelling equilibrium method), have been carried out in DDW, buffer solution of different pH (2.2, 6.8 and 7.4) and 0.9% of NaCl solution by gravimetric method according to reported procedure [30]. Briefly, known weight of dried polymers were placed in tea bags and dipped in an excess amount of the swelling media at 37 °C. After every 30 min, samples were removed from the various swelling mediums, excess surface solvent was carefully wiped with tissue paper, and water uptake was monitored on an electronic scale. All of the experiments were done in triplicate and reported as a mean of three readings. The swelling ratio (SR) and the degree of swelling or percentage swelling (P_S_) of swollen polymeric gel samples were evaluated as per Eq. 2.

2 $$ SR = \tfrac{{W_{t} - W_{o} }}{{W_{o} }}\quad {g/L} $$

where, *W*_*o*_ and *W*_*t*_represents initial dry weight and swollen weight of the hydrogels at fixed interval of time expressed in gram respectively.

The percentage swelling (P_S_) of the polymeric networks was calculated as:

3 $$ P_{s} \left( {\tfrac{{W_{t} - W_{o} }}{{W_{o} }}} \right) \times 100 $$

Swelling behavior of hydrogel has been investigated as a function of concentration of catechin, method of loading of catechin (in situ/swelling equilibrium method) in swelling medium. The power law expression proposed by Ritger and Peppas [40] was used to evaluate swelling kinetics:

4 $$ \frac{{W_{t} }}{{W_{\infty } }} = Kt^{n} $$

where, *W*_*t*_and *W*_*∞*_are the swollen weight of the hydrogels at fixed interval of time and equilibrium swelling expressed in gram respectively. ‘t’ is the time expressed in minutes, ‘n’ is the diffusion exponent and K is the diffusion constant.

By taking the natural log of Eq. (4), we get.

5 $$ \ln \frac{{W_{t} }}{{W_{\infty } }} = \ln (K) + n\ln (t) $$

The initial diffusion coefficient (D_i_), the average diffusion coefficient (D_A_) and late diffusion coefficient (D_L_) have been evaluated by using following equations.

6 $$ \frac{{W_{t} }}{{W_{\infty } }} = 4\left( {\frac{{D_{i} t}}{{\pi l^{2} }}} \right)^{{0.5}} $$

7 $$\:{\mathrm{D}}_{\mathrm{A\:\:}}\mathrm{\:=\:\:}\frac{\mathrm{0.049}{\mathrm{l}}^{\mathrm{2}}}{{\mathrm{t}}^{\frac{\mathrm{1}}{\mathrm{2}}}}$$

8 $$ \frac{{W_{t} }}{{W_{\infty } }} = 1 - \left( {\frac{8}{{\pi ^{2} }}} \right)\exp \left[ {\frac{{\left( { - \pi ^{2} tD_{L} } \right)}}{{l^{2} }}} \right] $$

where t^1/2^ is the time required for the 50% swelling and *‘l’* is the thickness of the sample.

### Drug loading to polymeric matrix

Loading of MTXinto functionalized gel matrixOFC and OFCC was carried out by swelling equilibrium method. Dried gel matrix (300 mg) ware immersed in 30 ml of drug solution (300 µg/ml). Hydrogelmatrix was allowed to swell for 24 h at room temperature in the drug solution. After stipulated time, MTX loaded hydrogels were carefully removed from the drug solution and washed with distilled water to remove the adsorbed drug on the surface of the hydrogels, followed by drying at room temperature to obtain the release device. Drug loaded gel matrix was designated as OFC-MTX and OFCC–MTX hydrogels respectively (Fig. 2 & Supplementary Fig. S4). Entrapment efficiency (%) of MTX in OFC and OFCC gel matrix were evaluated spectrophotometrically at λ max = 302 nm according to the calibration curve. The Drug Loading efficiency (%) of the drug was determined by using the following equation [41].

9 $$ {\text{Drug loading efficiency}}\left( \% \right) = \frac{{{\mathrm{C}}_{0} - {\mathrm{C}}_{{\mathrm{t}}} }}{{{\mathrm{C}}_{0} }} \times 100 $$

where C_0_ is the initial concentration (µg/ml) of the drug used and C_t_ is the unloaded concentration of the drug left in the solution after loading into polymeric hydrogel matrix.

### In vitro MTX drug release from polymeric matrix

The in vitro release studies of MTX from OFC-MTX and OFCC–MTX hydrogels have been evaluating by following the USP approved method (General Chapter < 711>, Dissolution, USP 27-United States Pharmacopeia Convention, Inc.,Rockville, MD) [42]. Drug loaded matrix (100 mg)was immersed in 20 ml release mediums of pH 2.2, 7, 7.4, simulated gastric fluid (SGF) of pH 1.2 and simulated intestinal fluid (SIF) of pH 6.8 at 37 °C under continuous stirring condition. After every 30 min, 5 ml of aliquots of solution was withdrawn followed by the addition of the same volume of fresh buffer solutions in order to keep the constant volume of the release medium. The amount of MTX released was measured spectrophotometrically by taking the absorbance at λ max=302 nm in all release mediums. All the experiments were performed in triplicates and reported as a mean of three readings. The cumulative release (R %) of the MTX from OFC-MTX and OFCC–MTX hydrogels had been evaluated using following equation [39].

10 $$ {\text{Cumulative release R}}\left( \% \right) = \frac{{{\mathrm{M}}_{{\mathrm{t}}} }}{{{\mathrm{M}}_{{\mathrm{o}}} }} \times 100 $$

where M_t_ and M_o_ are the drug released at time ‘t’ and initial loaded drug content respectively.

#### In vitro drug release kinetics and drug release mechanisms

The quantity of MTX released from OFC-MTX and OFCC-MTX hydrogels had been evaluated using different kinetics models viz.: zero order, first order, Higuchi’s and Korsmeyer-Peppas models (Eqs. 11, 12, 13, 14) to characterize drug release mechanism MTX from cross-linked polymeric hydrogels [40].

11 $$ {\mathrm{C}} = {\mathrm{K}}_{{\mathrm{o}}} {\mathrm{t}} $$

12 $$ \ln {\mathrm{C}} = \ln {\mathrm{C}}_{{\mathrm{o}}} - {\mathrm{K}}_{{\mathrm{1}}} {\mathrm{t}} $$

13 $$ {\mathrm{M}}_{{\mathrm{t}}} /{\mathrm{M}}_{\infty } = {\mathrm{K}}_{{\mathrm{H}}} {\mathrm{t}}^{{{\mathrm{1}}/{\mathrm{2}}}} $$

14 $$ {\mathrm{M}}_{{\mathrm{t}}} /{\mathrm{M}}_{\infty } = {\mathrm{Kt}}^{{\mathrm{n}}} $$

By taking the natural log of Eq. (14)

15 $$ ln{\mathrm{M}}_{{\mathrm{t}}} /{\mathrm{M}}_{\infty } = ln\left( {\mathrm{K}} \right){\text{ }} + {\mathrm{n}}ln\left( {\mathrm{t}} \right) $$

where C, C_o,_ K_o_, K_1_, K_H_, M_t_, M_∞_, M_t_/M_∞_, K, n and R^2^ are % cumulative drug release at time t, initial concentration of the drug, zero order release rate constant, first order release rate constant, Higuchi constant, cumulative fraction of drug released during time (t), cumulative fraction of drug released at equilibrium, fraction released drug during time (t), Gel characteristic constant of the drug-polymer system, diffusion exponent. The value of ‘n’ is used for characterizing the mechanism of drug released. The value of ‘n’=0.5 correspond to normal Fickian diffusion mechanism which is a diffusion-controlled mechanism. The value of ‘n’ between 0.5 < n < 1 corresponds to non Fickian diffusion mechanism where diffusion rate and rate of polymer chain relaxation are comparable. In case value of ‘n’$\:\ge\:$1, case-II diffusion mechanism is followed where rate of diffusion is rapid as compared to the rate of polymer chain relaxation.D_i_, D_A_ and D_L_ for drug releasedfrom hydrogel networks had been evaluated by using the following equations.

16 $$ \frac{{M_{t} }}{{M_{\infty } }} = 4\left( {\frac{{D_{i} t}}{{\pi l^{2} }}} \right)^{{0.5}} $$

17 $$ {\mathrm{D}}_{{\mathrm{A}}} {\text{ = }}\frac{{{\mathrm{0}}{\mathrm{.049l}}^{{\mathrm{2}}} }}{{{\mathrm{t}}^{{\frac{{\mathrm{1}}}{{\mathrm{2}}}}} }} $$

18 $$ \frac{{M_{t} }}{{M_{\infty } }} = 1 - \left( {\frac{8}{{\pi ^{2} }}} \right)\exp \left[ {\frac{{\left( { - \pi ^{2} tD_{L} } \right)}}{{l^{2} }}} \right] $$

where, M_t_/M_∞_ is the fraction release of the drug, M_t_ is the release of the drug at time, t,M_∞_ is the release of drug at equilibrium, t ^1/2^ is the time required for the 50% release of the drug.

### Evaluation of biological properties of OFC and OFCC matrix

Blood compatibility, thrombogenicity and haemolytic potential of pristine natural gum, OFG, OFC and OFCC hydrogel matrix were determined as per the procedure mentioned in literature [43]. Antioxidant activity of catechin extracted, OFC and OFCC networks (2500µg/ml and 5000 µg/m) were evaluated by DPPH [2, 2’-diphenyl-1-picrylhydra] radical scavenging method [44]. Hydrolytic degradation studies of FG, OFC and OFCC bio-nanocomposite hydrogels were carried in PBS buffer pH 7.4 by gravimetric method [45]. Each set of experiment have been performed in triplicate.

#### Thrombogenic behaviour of functionalized gel

The thrombogenicity of a freshly synthesised gel matrix was assessed to determine its preliminary biological compatibility. To assess thrombus formation on polymeric surfaces, the gravimetric approach [46, 47] based on the weight of the blood clot formed during the interaction of blood and polymeric surface was adopted. Briefly, hydrogel samples (0.01 mg) were dipped in small amount of the phosphate buffer saline (PBS) of pH 7.4 and kept it overnight at 37^o^Cbefore testing. On next day, PBS solution was decanted out and replaced with acid citrate dextrose (ACD) blood (1 ml) and 0.1 M CaCl_2_ (0.025 ml) (US Pharmacopeia XXIII, 1994) in each sample bottles and were maintained undisturbed for 50–55 min. To prevent clotting, DDW (10 ml) was added to the reaction system after the specified time. The generated thrombus was removed using a spatula and immersed in DDW (5–10 ml) for 5 min before being fixed with 3 ml formalin (37% formaldehyde). The thrombus was blotted with basic filter paper, dried at 50 °C, and weighed. The glass beaker without sample and glass beaker without sample and blood were taken as positive and negative control respectively. The thrombus % was calculated by following formula given below:

19 $$ {\mathrm{Thrombose}}\left( \% \right) = \frac{{{\text{Mass of the test sample}} - {\text{Mass of }}\left( - \right){\mathrm{control}}}}{{{\text{Mass of }}\left( + \right){\mathrm{control}} - {\text{Mass of }}\left( - \right){\mathrm{control}}}} \times 100 $$

#### Haemolytic potential of functionalized gel

Hemolytic potential ofFG, OFG, OFC and OFCC bio-nanocomposite gel matrix were evaluated as per the procedure described in American Society for Testing and Materials (ASTM) [48]. The samples were kept in PBS buffer (pH 7.4) for 24 h at 37 °C. To each sample, ACD blood (1 ml) was added and reaction system was maintained at 37 °C for 3 h. For positive control and negative control ACD blood (1 ml) was added to DDW (7 ml) and PBS buffer (7 ml), respectively. To ensure that the samples made contact with the blood, each tube was gently inverted three times. Each tube was incubated and then centrifuged at 10,000 rpm for 10 min. The hemoglobin released by haemolysis was evaluated by measuring the absorbance of the supernatant of each sample at λ max = 540 nm using UV spectrophotometer (Elico, SL-164). The haemolysis % was calculated by using following formula.

20 $$ {\mathrm{Haemolysis}}\left( \% \right) = \frac{{{\text{Absorbance of sample}} - {\mathrm{Absorbance}}( - ){\mathrm{control}}}}{{{\text{Absorbance }}\left( + \right){\mathrm{control}} - {\mathrm{Absorbance}}( - ){\mathrm{control}}}} \times 100 $$

#### Antioxidant activity of functionalized gel

**Fig. 3:**
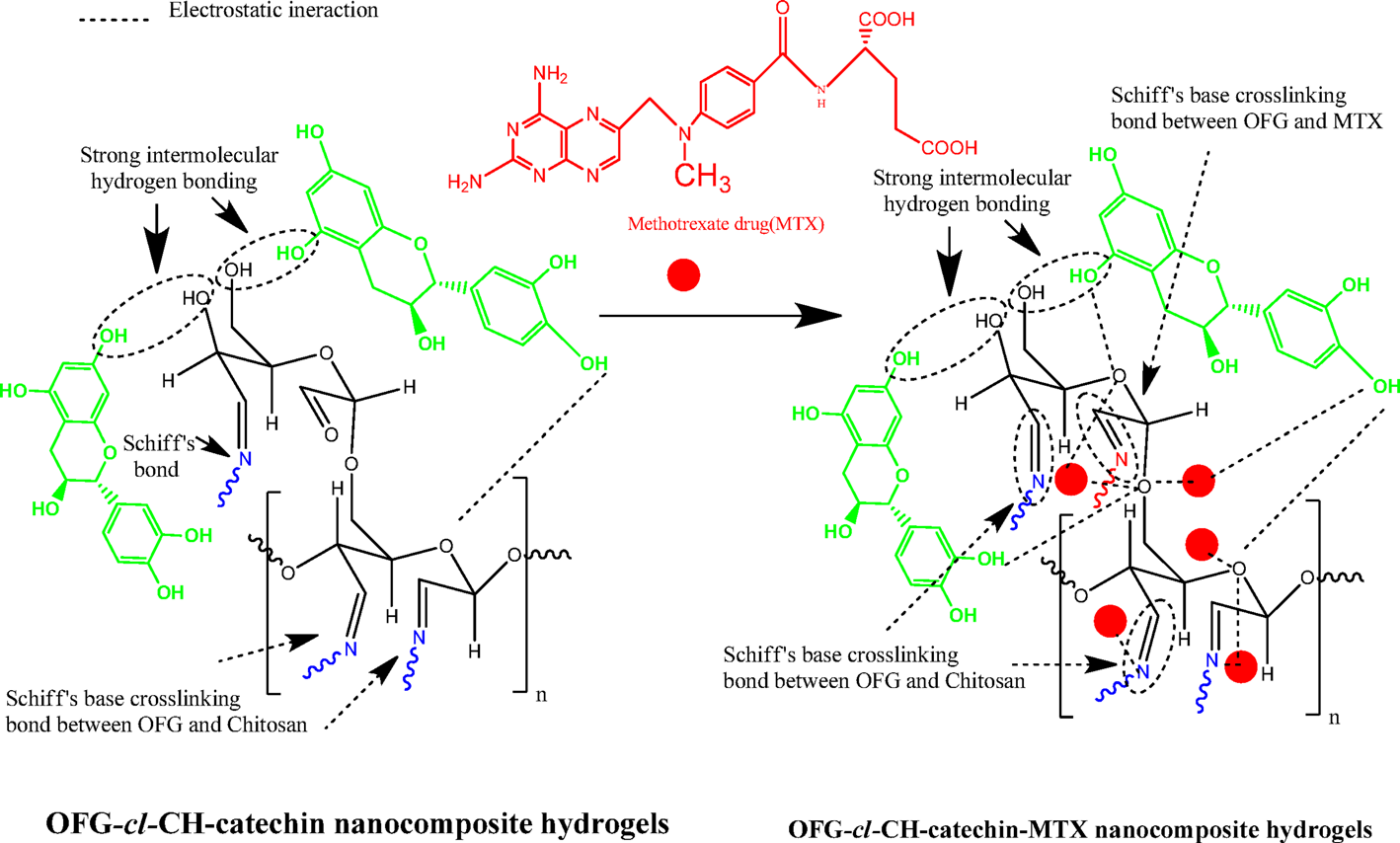
Plausible mechanism and schematic representation of loading of model drug MTX in OFG-*cl*-CH-catechin nanocomposite hydrogel

DPPH (2,2-diphenyl-1-picrylhydrahyl) is a widely used bioanalytical method for determining the antioxidant capacity of herbal extracts and polyphenolic compounds. It involves combining antioxidant compounds with DPPH solution and evaluating absorbance of the resultant solution after stipulated interval of time. When combined with H-donor bioactive chemicals, the DPPH radical transforms into a pale-yellow DPPH due to the reduction of a single electron on the nitrogen atom in DPPH radical to corresponding hydrazine by removing the H-atom from the antioxidant ((Fig. 3 (a–b)) [49].

In presentstudy, the antioxidant properties of the catechin, OFC, and OFCC networks were assessed using the previously published DPPH radical scavenging assay technique, with minor changes [44]. Briefly, 4 mg of each sample (catechin, OFC, and OFCC) and 0.004% of DPPH (in methanol) was vortexed and incubated in the dark at 30 °C for 45 min. The absorbance of each sample was measured with a UV-Vis spectrophotometer (Elico, SL-164) at 517 nm.Each sample was examined three times. The DPPH radical scavenging activity of each sample was measured using the following formula.

21 $$ {\text{DPPH radical scavenging activity}}\left( \% \right) = \frac{{{\mathrm{Ao}} - {\mathrm{A}}1}}{{{\mathrm{Ao}}}} \times 100 $$

where, A_0_ and A_1_were the absorbance of blank and absorbance of each tested sample.

#### Hydrolytic degradation of functionalized gel

The hydrolytic degradation behaviour of the FG, OFG, OFC, and OFCC nanocomposite hydrogels was examined in vitro in PBS at pH 7.4 gravimetrically for one month duration [50]. Briefly, known pre-weighted dry hydrogel samples were soaked at 37 °C in pH 7.4 PBS (30 ml). Initially, swelling caused an increase in the weight of hydrogels. Every day, the media was replaced to ensure that all degraded or dissolved components were removed.

The dipped samples were taken out at regular intervals (7, 15, and 30 days), rinsed with DDW, air-dried, and weighed. The hydrolytic degradation tests were carried out three times, and the average data were taken into consideration. Degradation % was calculated by the following formula.

22 $$ {\mathrm{Degradation}}\left( \% \right) = \frac{{{\text{Initial weight}} - {\text{Final weight}}}}{{{\text{Initial weight}}}} \times 100 $$

A graph was plotted between weight loss (%) vs. time (days), in order to analyze the comparative analysis of different hydrogels samples.

## Results and discussion

### Isolation, phytochemical profiling, and chromatographic characterization of catechin from *Acacia catechu* heartwood

Previous studies have stated that the heartwood of *Acacia catechu* has a content of 3.30% catechins [51]. The traditional techniques for extracting catechins required a number of processes and were not profitable. In present study, catechin was extracted from heartwood of *Acacia catechu* plant usinga green technique which is more economic and convenient. The catechin extraction procedure was already elaborated in Sect. 2.2. Dry crude water extract of *Acacia catechu* (4 g) was extracted with ethyl acetate as organic medium and up to 2.30% yield of catechin was achieved. Catechin thus extracted was characterized by LC/MS analysis, HPLC method, Powder XRD and FTIR and results are found in good agreement with the literature reported. Plant polyphenols have antioxidant properties due to their phenolic content, which scavenges free radicals and produces singlet oxygen, hydroxyl radicals [52].

Preliminary quantitative tests of catechin had been carried out as per standard tests and had shown presence of phytochemicals viz. phenols, alkaloids, steroids, flavonoids, carbohydrates and terpenoids. The results were represented as + (Present) or –(Absent) which indicated the presence or absence of particular classes of compounds (Supplementary Table S1 (b)).

Catechin extract was analysed using LC-MS, and catechin metabolites isolated were identified by comparing retention time and m/z values to known standards. Epicatechin is an epimer of catechin with the same mass spectrum as catechin. In the current work, LC-MS analysis of extracted catechin displays catechin and epicatechin peaks matching the molecular ion at m/z 291.10 and 139.05, along with retention duration and intensity at 6.07 min (100% intensity) and 7.56 min (47% intensity) respectively under a 20% collision energy level (Supplementary Fis. S5 and S6). It was further reported that fragmentation peaks at m/z 291 and m/z 289–292 correspond to the catechin unit [38], while peaks at m/z 291.2 and m/z 139.3 corresponds to epicatechin [53]. In present study, 0.1% formic acid was used to protonate catechin and +ve ion mode was opted due to its higher sensitivity compared to the -ve ion mode. This method was chosen due to the expected ionization of most bioactive chemicals in this mode.

HPLC was employed to detect and quantify bioactive components (catechin and epicatechin) in a mixture extracted from *Acacia catechu* plant heartwood. Both components were quantified by comparing the retention time (RT) of HPLC chromatograms with that of standard catechin and epicatechin of previously reported HPLC chromatograms from the literature [54]. HPLC chromatogram clearly demonstrated peaks of catechin and epicatechin (Supplementary Fig. S7 (a–b)). The plant extract had a steady retention time for both catechin and epicatechin, which was equivalent to the RT of standards. HPLC results are shown in supplementary Table S1 (c).A similar study had demonstrated that *Acacia catechu* plant extract contains a high concentration of catechin and epicatechin [55].

### Synthesis of OFG-*cl-*CH/Catechin hydrogel and encapsulation efficiency of catechin in OFG*-cl*-CH (Schiff base)

Catechin, a natural polyphenol, has limited absorption and bioavailability due to its low solubility and weak stability. By protecting against metabolism and degradation, catechin’s bioavailability could be improved, potentially increasing its medicinal efficacy. In this study, OFG*-cl*-CH (OFC) nanocomposite hydrogel wassynthesized by Schiff’s base reaction between active –NH_2_ groups of chitosan and –CHO groups of OFG where OFG act as natural cross-linking agent. Sodium periodate selectively oxidise vicinal hydroxyl groups on FG backbone to carbonyl groups (Fig. 1 & Supplementary Fig. S3).Aldehydic groups generated on OFG facilitates cross-linking with the free –NH_2_ groups of chitosan to form Schiff’s base (-RC = N) without using any toxic/synthetic initiator and cross-linker system. OFC then further functionalized with catechin to form OFG-*cl*-CH-catechin (OFCC) polymeric hydrogels. Figure 1(b) represented plausible interaction between catechin and OFC hydrogel network. Catechin and Schiff’s base hydrogel may form strong intermolecular hydrogen bonds. Unreacted OH groups on OFC served as a location for end-to-end hydrogen bonding with catechin’s OH terminal groups. Sarika et al. had found that in curcumin loaded gum arabic aldehyde-gelatin matrix, there wassubstantial intermolecular hydrogen bonding between the unreacted -OH groups of gum arabic aldehydic and hydroxyl groups of curcumin responsible for effective encapsulation of polyphenol [8, 12]. In present work, catechin’s encapsulation efficiency up to 53.23% was achieved using the swelling equilibrium method and the resultant gel (OFCC) becomes yellowish brown in colour. Dark colored gel matrix was formed when entrapment of catechin was done via in situ technique. The color of the gel matrix intensifies from brown to dark brown with rise in catechin concentration (Supplementary Fig. S2). Gel matrix synthesized through in situ method showed higher swelling percentage than that synthesized via swelling equilibrium method. Swelling decreased with rise in concentration of encapsulated catechin. Therefore, OFCC with 2500 µg/ml catechin synthesized via in situ technique was chosen as the optimized gel matrix for subsequent drug release studies.

### Characterization of functionalized gel matrix

#### X-ray analysis

**Fig. 4:**
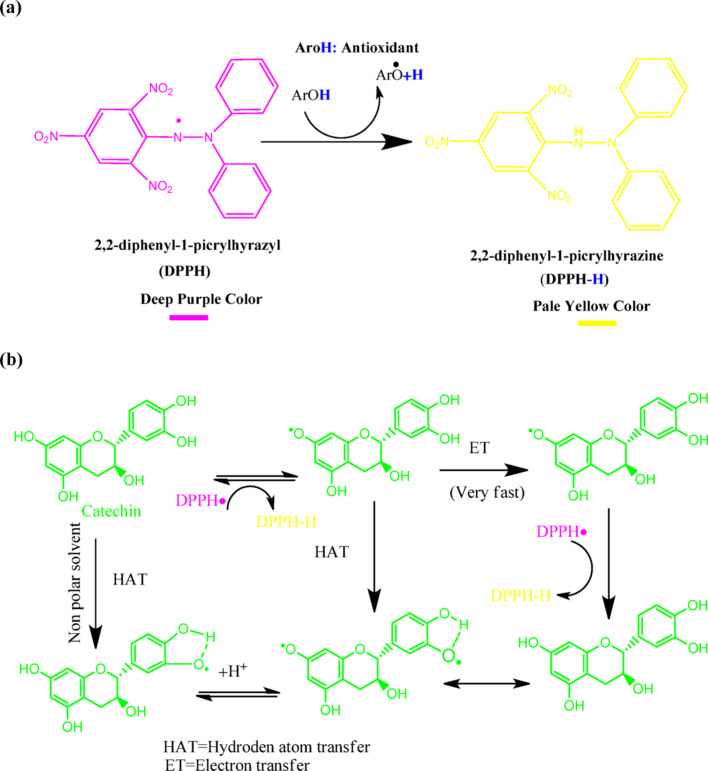
**a**,** b**: Antioxidant study using DPPH method: (**a**) Reaction mechanism of DPPH free radical with an antioxidant; (**b**) Reaction mechanism of DPPH free radical with Catechin (extracted from *Acacia catechu*: Present case)

**Fig. 5:**
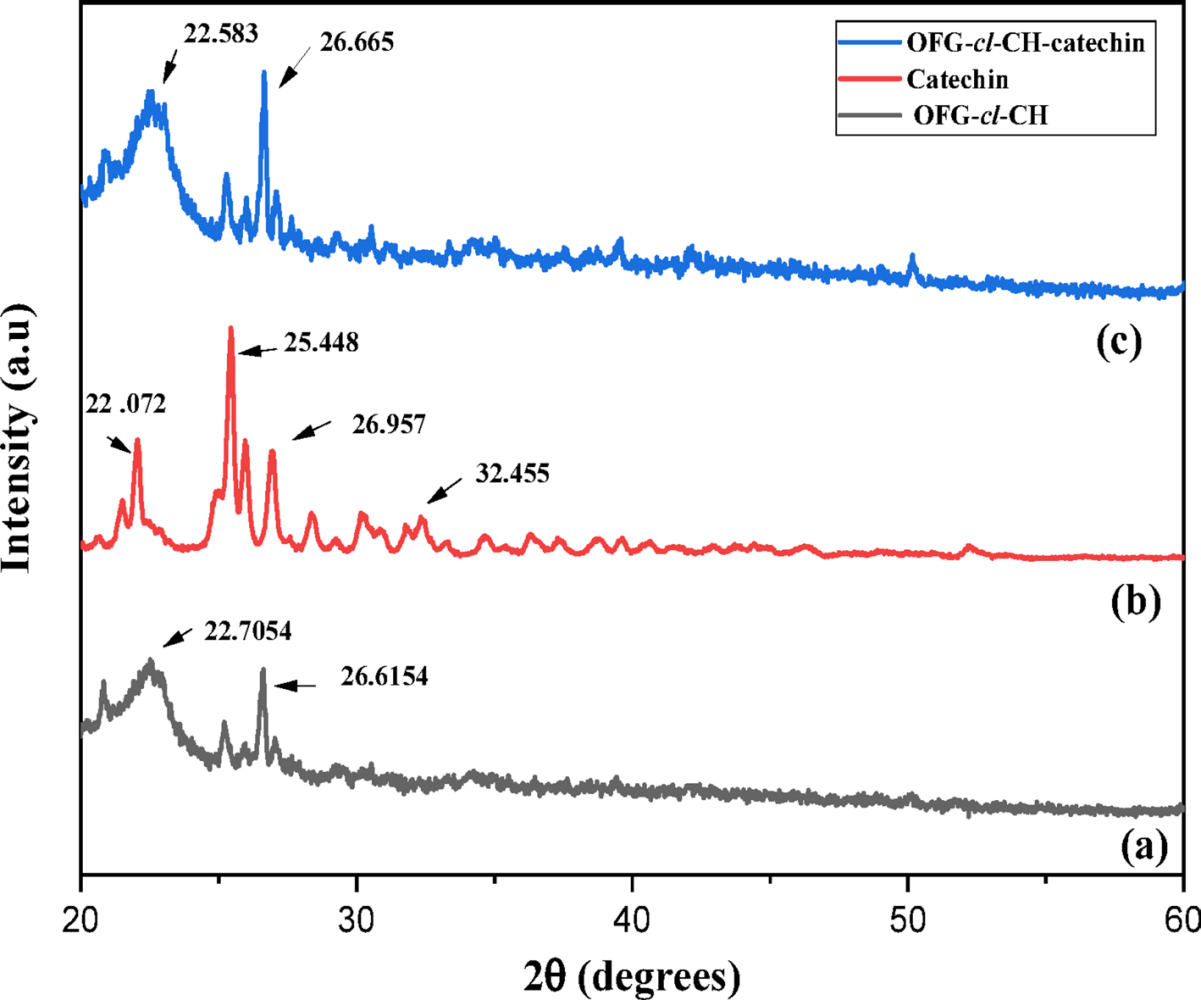
Powdered XRD spectra of** a** OFC gel matrix [57];** b** Catechin;** c** OFCCnanocomposite gel matrix

X-ray diffraction pattern of OFC, catechin, OFCC hydrogel were recorded and results are presented in Fig. 4 (a–c). Powder XRD analysis of MTX loadedgel matrix viz.: OFG*-cl*-CH(OFC-MTX) andOFG*-cl*-CH-catechin (OFCC-MTX) was performed and results are shown in Fig. 5(a–b).XRD analysis of catechin revealed crystalline peaks at 2θ = 22.072°, 25.448°, 26.957°, and 32.455 ° (Fig. 4, curve b). The XRD spectrum of OFCC hydrogel (Fig. 4, curve c) shows two peaks at 2θ = 22.583° and 2θ = 26.665°, which are identical to OFC [28]. XRD spectra clearly depicted the incorporation of catechin in the gel matrix with distinct peak at around 2θ = 25°, which is characteristic peak of catechin apart from other peaks. In another study, catechin-g-chitosan conjugate had shown that catechin incorporation resulted in a crystalline area in the 10–50° range [56], indicating that catechin boosted the crystallinity of matrix.

As per literature survey, XRD peaks of MTX were observed at 13.7°, 14.1°, 19.6°, 27.8°, and 29° whichattributed to crystalline nature of drug molecules [16]. In present study, XRD spectrums of OFC-MTX (Fig. 5, curve a) and OFCC-MTX (Fig. 5, curve b) have broad peak at 2θ = 22.558° and 2θ = 22.174° respectively. There was shifting and broadening of peaks in functionalized matrix as compared to characteristic peaks of MTX, which was caused by the interaction of natural catechin nanoparticles with MTX molecules within the polymeric backbone and these results validated the loading of MTX in OFC and OFCC.

Average crystalline size of embedded catechin nanoparticles in OFCC and OFCC-MTX hydrogel matrix had been evaluated from their respective powder XRD spectrum using Scherrer’s equation (Eq. 23) [51].

23 $$ D = \frac{{K\lambda }}{{\beta \mathrm{Cos} \theta }} $$

where D (nm), λ (nm) = 1.54A^o^,β (radian) and θ (radian) are crystalline size, wavelength, full wave at half maximum and Bragg’s angle respectively. K is constant with value of 0.94. It had been observed that average particle size of free catechin nanoparticles and catechin incorporated in OFCC and OFCC-MTX hydrogels were found 34 nm, 22 nm and 21 nm respectively.

#### FTIR analysis

**Fig. 6:**
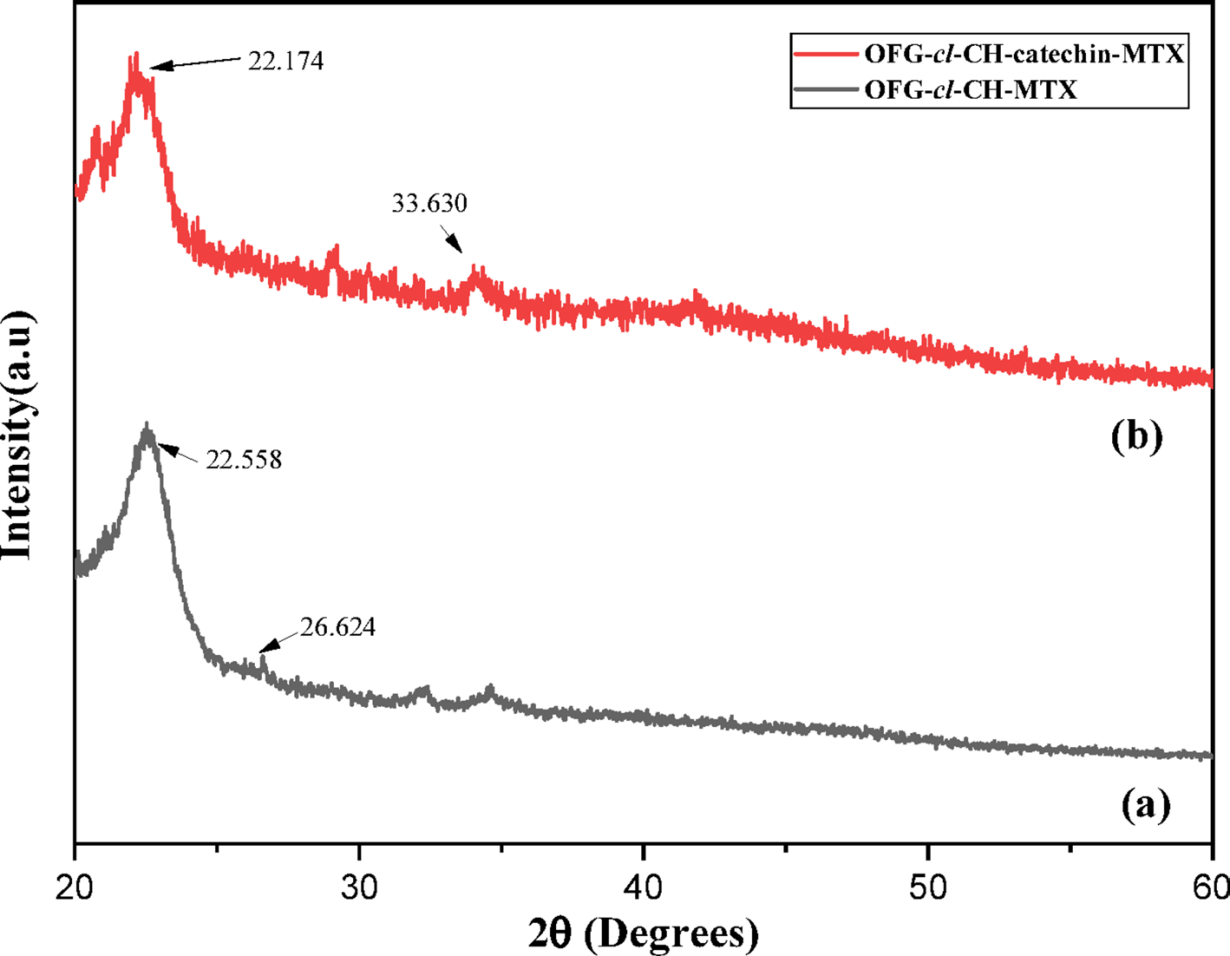
PowderedXRD spectra of** a** OFC-MTXgel matrix;** b** OFCC-MTXnanocomposite gel matrix

In present work, FTIR analysis of freshly extracted catechin, OFC and OFCC synthesized using in situ and swelling equilibrium methods had been recorded to confirm the structural functionalization Fig. (6). FTIR spectra of catechin had shown broad band around 3200–3450 cm^− 1,^ band at 2925 cm^− 1^, are attributed to OH groups and C-H stretching and methylene (-CH_2_) groups of polyphenol backbone respectively. Absorption peak at 1864 cm^− 1^ was might be due to coordination bonding of aromatic ring –OH groups. Aromatic ring vibrational peaks had also been recorded at 1620 cm^−1^and 1525 cm^− 1^ and peak at 1473 cm^−1^was due to aliphatic CH_2_ bending vibration. Apart from these, some characteristic absorption bands were also observed at 970 cm^− 1^, 1035 cm^−1^and 1149 cm^−1^which were characteristic bands for C-H bending vibration and C-O stretching of alkene and aromatic moiety of catechin (Fig. 6b) [58].

**Fig. 7:**
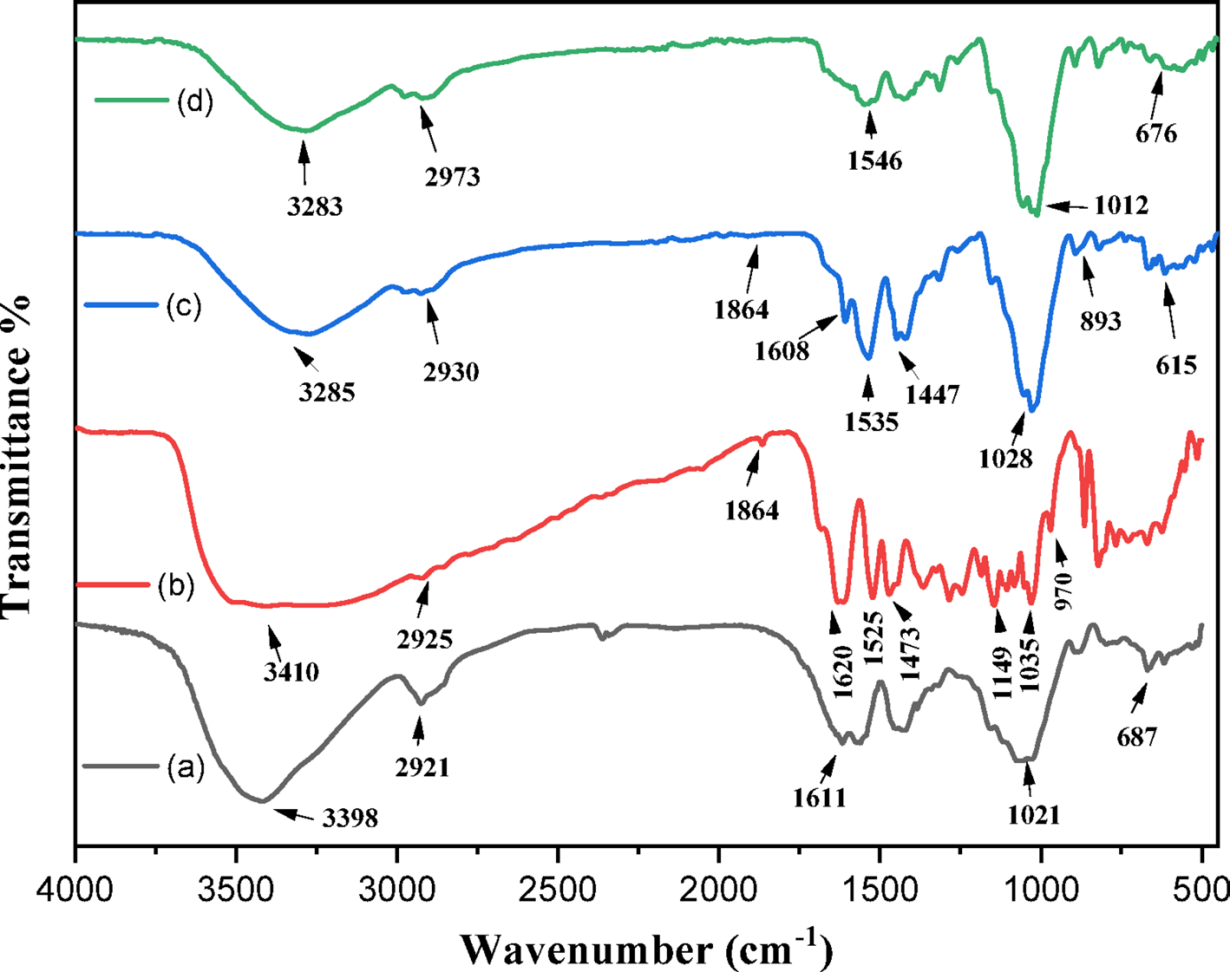
FTIR spectra of** a** OFCgel matrix [59];** b** Catechin;** c** OFCC nanocomposite gel matrix*(in-situ* method);** d** OFCC nanocomposite gel matrix (swelling equilibrium method)

FTIR spectra of OFCC had shown characteristic peaks of polysaccharide backbone as well as catechin embedded (Fig. 6c). Peak at 3272 cm^−1^was attributed to the presence of intermolecular hydrogen bonding between –OH groupsof catechin and unreacted –OH groups of the OFC. Peak at 1608 cm^−1^correspond to aromatic C = C stretching of catechin (Curve c). This peak was overlapped by the –C = N imine bond stretching peak of OFCmatrix (Fig. 6d). There was also appearance of strong bands at 1535 cm^− 1^ and 1546 cm^− 1^ which correspond to C-C stretching of aromatic rings of catechin embedded [9]. According to literature survey, FTIR spectra of MTX exhibits peaks at 3420 cm^− 1^, 1670 cm^−1^and 1600 cm^− 1^, which correspond to O-H, C = O and N-H stretching, respectively. Absorption bands in 1400 –1200 cm^−1^correspond toamide group stretching [51]. In FTIR spectra of drug-loaded OFC-MTX and OFCC-MTX, characteristic peaks of MTX loaded had been observed as merged with peaks of other functional groups of polymeric backbones (Fig. 7(a–b). A medium intensity peak at 1640 cm^−1^in OFC-MTX was attributed to stretching of carbonyl groups of MTX. Due to incorporation of MTX, characteristics absorption bands of OFC and OFCC at around 2921 cm^− 1^, 1610–1611 cm^− 1^ had also been shifted towards higher wave number i.e. 2968 cm^− 1^, 1640 cm^− 1^ in OFC-MTX and 2933 cm^− 1^, 1640 cm^− 1^ OFCC-MTX respectively. These findings suggested that drug molecules might interacted through intermolecular interactions within the gel matrix. Since no new absorption bands appeared in drug loaded matrix which confirmed that there was no chemical reaction between the drug and the polymeric hydrogels and indicated that MTX is embedded in the gel matrix with secondary interactions only.

#### Thermogravimetric analysis

**Fig. 8:**
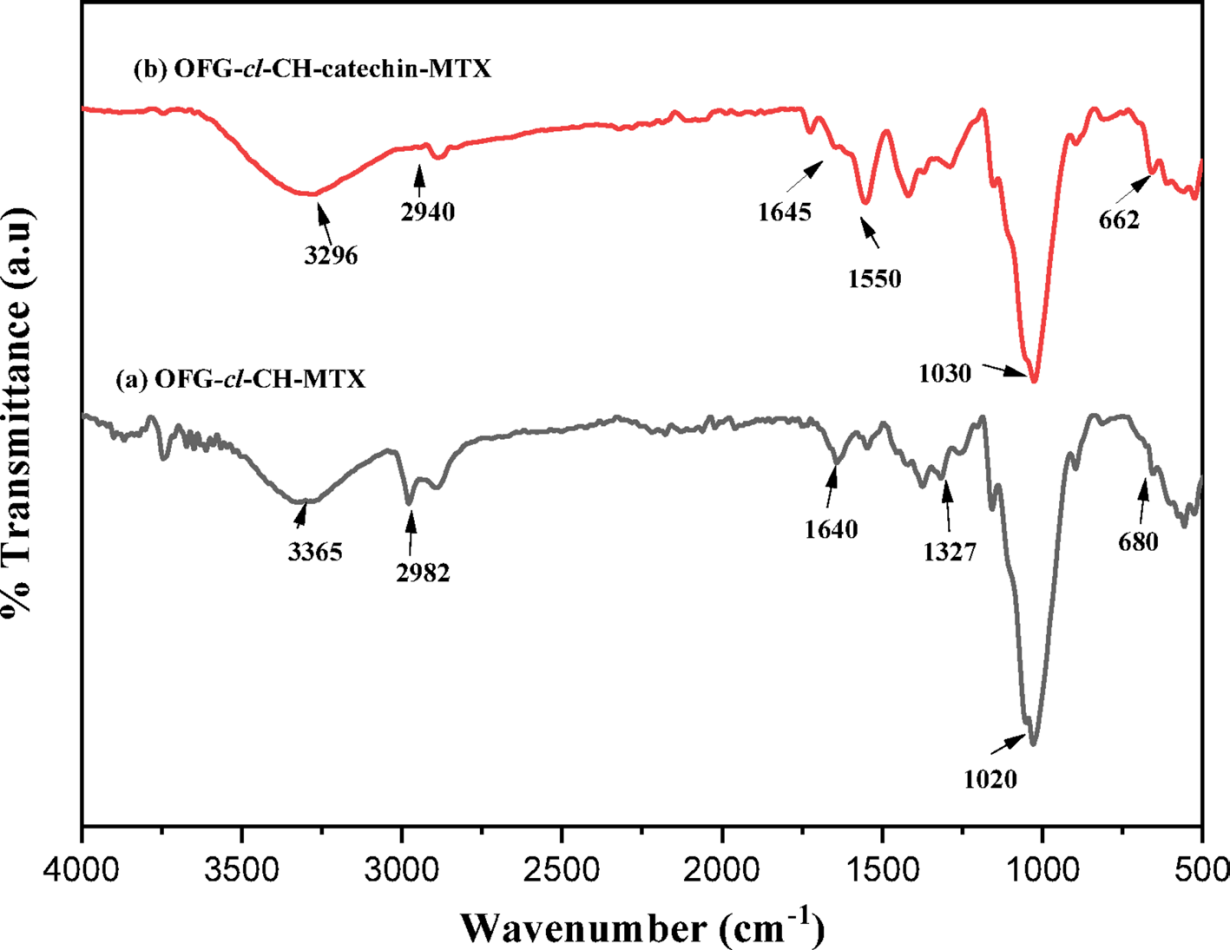
FTIR spectra of** a** OFC-MTXgel matrix;** b** OFCC-MTXnanocomposite gel matrix

Thermal behaviour of FG, OFG, OFC and OFCC polymeric matrix was evaluated through TGA analysis and results had been presented in Fig. 8. Initial decomposition temperature (IDT), final decomposition temperature (FDT) and decomposition temperature (DT) per 10% weight loss had been evaluated (Supplementary Table S2). IDT was the temperature at which polymeric decomposition was started and the weight loss was mainly due to evolution of retained moisture. IDT has been observed at 146.85 °C, 134.43 °C, 110.46 °C, 150.90 °C with respective residueupto 16, 9, 11.5, and 15%, respectively for FG, OFG, OFC, and OFCC. FDT had been observed at 507.25 °C (3.50% residue), 558.80 °C (1.10% residue), 505.91 °C (4.30% residue), 575.73 °C (1.10% residue) respectively for FG, OFG, OFC and OFCC matrix.

It was observed that during initial thermal decomposition stage, OFC matrix was thermally labile as compared to native FG. Multiple decomposition stages had been observed in all polymeric matrix. 1st and 2nd decomposition stages for FG and OFG had been observed at 195.27 °C (80% residue), 332.2 °C (19% residue) and 150.56 °C (86.87% residue), 346.56 °C (16.30% residue) respectively. These results described that oxidation make native gum more labile for decomposition.

In case of OFC, 1st and 2nd decomposition stage was observed at 143.31 °C (86.52% residue) and 429.29 °C (11.09% reside) whereas in case of OFCC, 155.05 °C (80% residue) and 494 °C (6% residue) were 1st and 2nd decomposition temperatures respectively. It was observed that functionalization of OFG with chitosan and further with catechin results in improvement in thermal stability of the OFC and OFCC gel matrix. OFCC matrix had acquired even greater stability than OFC which might be due to incorporation of catechin nanoparticles within the gel matrix which provided extra secondary interactions with in the gel matrix. Maroufiand coworkers had observed similar thermal behavior in Chitosan/dialdehyde guar gum incorporated with pomegranate peel extract-based gel matrix [60]. Catechin incorporation modifies OFC hydrogel networks structure by acting as a green cross-linkers, which increases cross-linking density and improves the physical & mechanical strength (stiffness). Therefore, catechin enhance the interfacial interactions through strong intermolecular hydrogen bonding between unreacted OH groups of OFC and catechin’s OH terminal groups resulting in improved structural stability, self-healing, controlled and stimuli-responsive release as well.

#### FESEM analysis

**Fig. 9:**
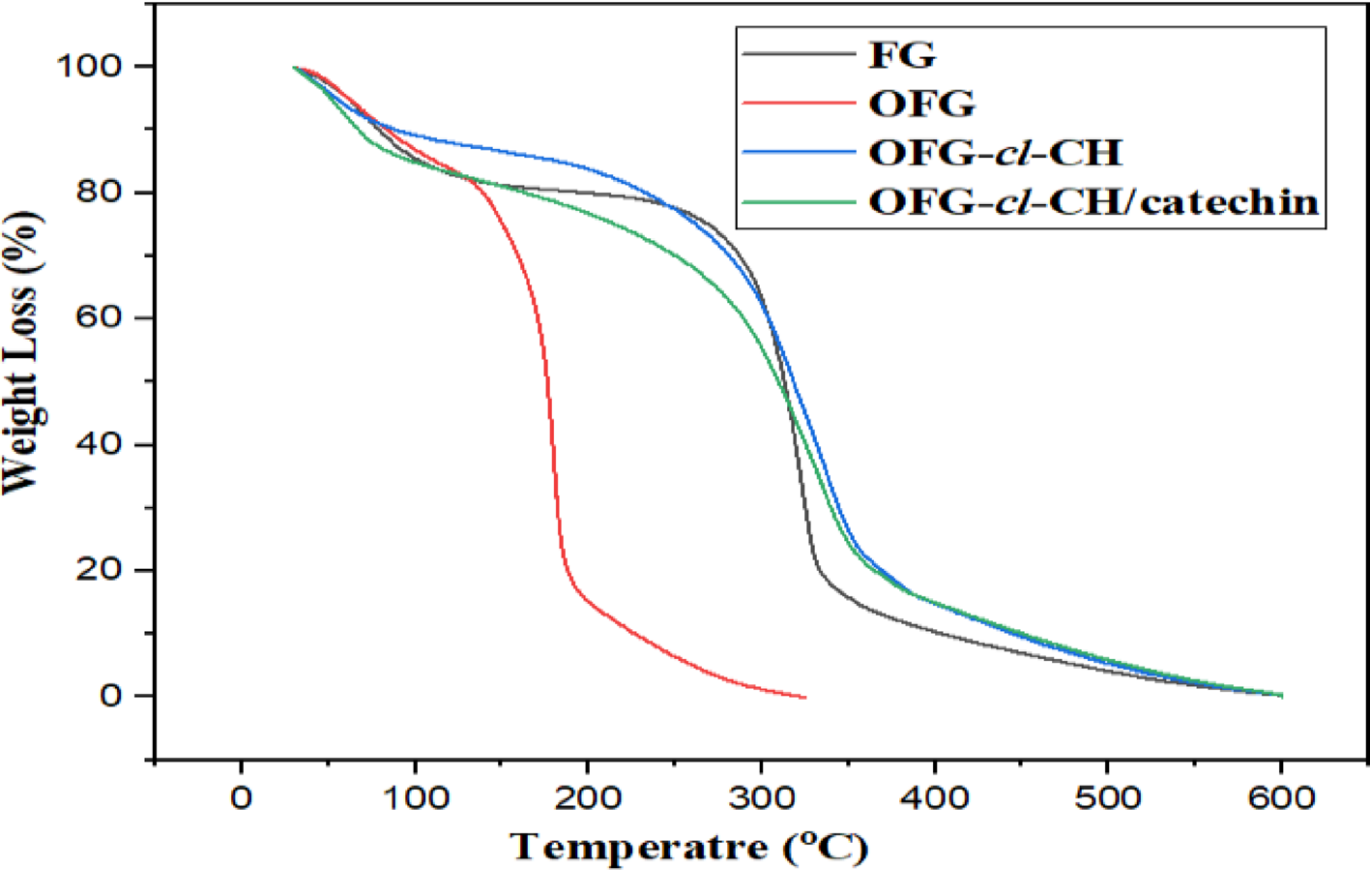
TGA of FG, OFG, OFC and OFCC gel matrix

FESEM is an advanced technology used to visualize the surface morphology and three-dimensional structure of functionalized gel networks. FESEM analysis of FG, OFG, OFG-*cl*-CH (OFC) had already been reported in our previous work (Fig. 9 (a–b)). Functionalization resulted in rough and uneven surface morphology of OFC [29]. In current study, FESEM of OFCC (Fig. 9(c–d) and drug incorporated gel matrix (Fig. 9(e–h) had been analyzed.

**Fig. 10:**
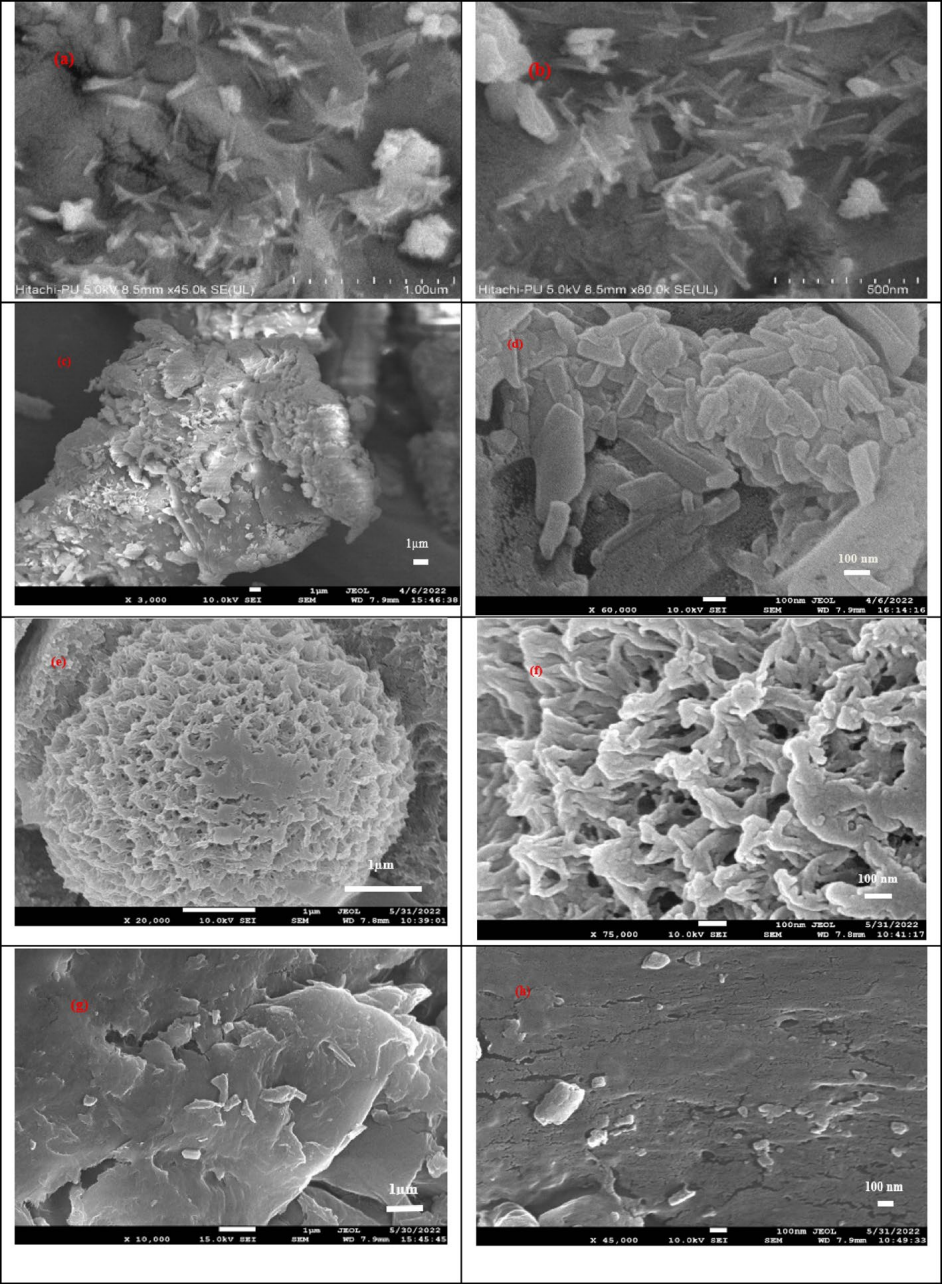
FESEM of** a**,** b** OFC gel matrix [57];** c**,** d** OFCC nanocomposite gel matrix;** e**,** f** OFC-MTX gel matrix;** g**,** h** OFCC-MTXnanocomposite gel matrix.Magnification: (**a**) × 45,000 (**b**) × 80,000 (**c**) ×3000 (**d**) ×60,000 (**e**) × 20,000 (**f**) × 75,000 (**g**) ×10,000 and (**h**) ×45,000

Incorporation of catechin in OFC gel resulted in smooth topography attributed to the formation of hydrogen bonding between catechin and OFC gel matrix (Fig. 10(c–d) [33]. Entanglement of catechin in gel matrix resulted in three-dimensional interconnected smooth and porous structures. Needle-like crystals observed might also appear due to catechin incorporated [37].

Loading of drug molecules also modify the surface morphology of the gel matrices. In present case, incorporation of MTX in both gel matrix viz.: OFC and OFCC nanocomposite drastically modify the surface morphology. There was appearance of large microspores with coarse structure throughout gel matrix (Fig. 9 (e-f)). In OFC-MTX, there were significantly visible pores in hydrogel matrix because of lesser drug loading [60] whereas in OFCC-MTX matrix, surface morphology of matrix was transformed from rough to smooth surface, which might be due to incorporation of catechin nanoparticles as well as drug molecules in the gel matrix facilitate more ionic interactions thus making the gel matrix more compact which further supported the efficient drug loading as well as incorporated catechin molecules (Fig. 9(g–h)).

#### TEM analysis

In present study, presence of catechin nanoparticles inside OFCC nanocomposite hydrogel matrix and their respective crystallite size had been investigated using TEM analysis (Fig. 10 (a–c)).It had been observed that, there was appearance of relatively spherical shape of catechin nanoparticles within the OFCC nanocomposite matrix. The average crystallite size of the catechin nanoparticles was approximately 30.68 nm and these results were in correlation with results obtained from powdered X-ray diffraction method using Scherrer’s formula (Eq. 23). The difference in shape was due to pore size variation in gel matrix. Most of embedded catechin nanoparticles dispersed uniformly and were spherical in shape throughout the gel matrix. There is no aggregation inside the produced hydrogel networks as depicted from particles size distribution plot.Mechanistically, the entrapment of catechin into OFC matrix occurs through a entanglement of functional groups of base polymers of OFC formation and physical interaction between free OH groups of OFC and catechin. This catechin embedded network structure with secondary interactionsmight also provides stability to drug loaded OFCC against premature release in neutral condition but allows for triggered release in acidic environment.

### Swelling kinetics of OFG-*cl*-CH-catechin (OFCC) nanocomposite hydrogel matrix

**Fig. 11:**
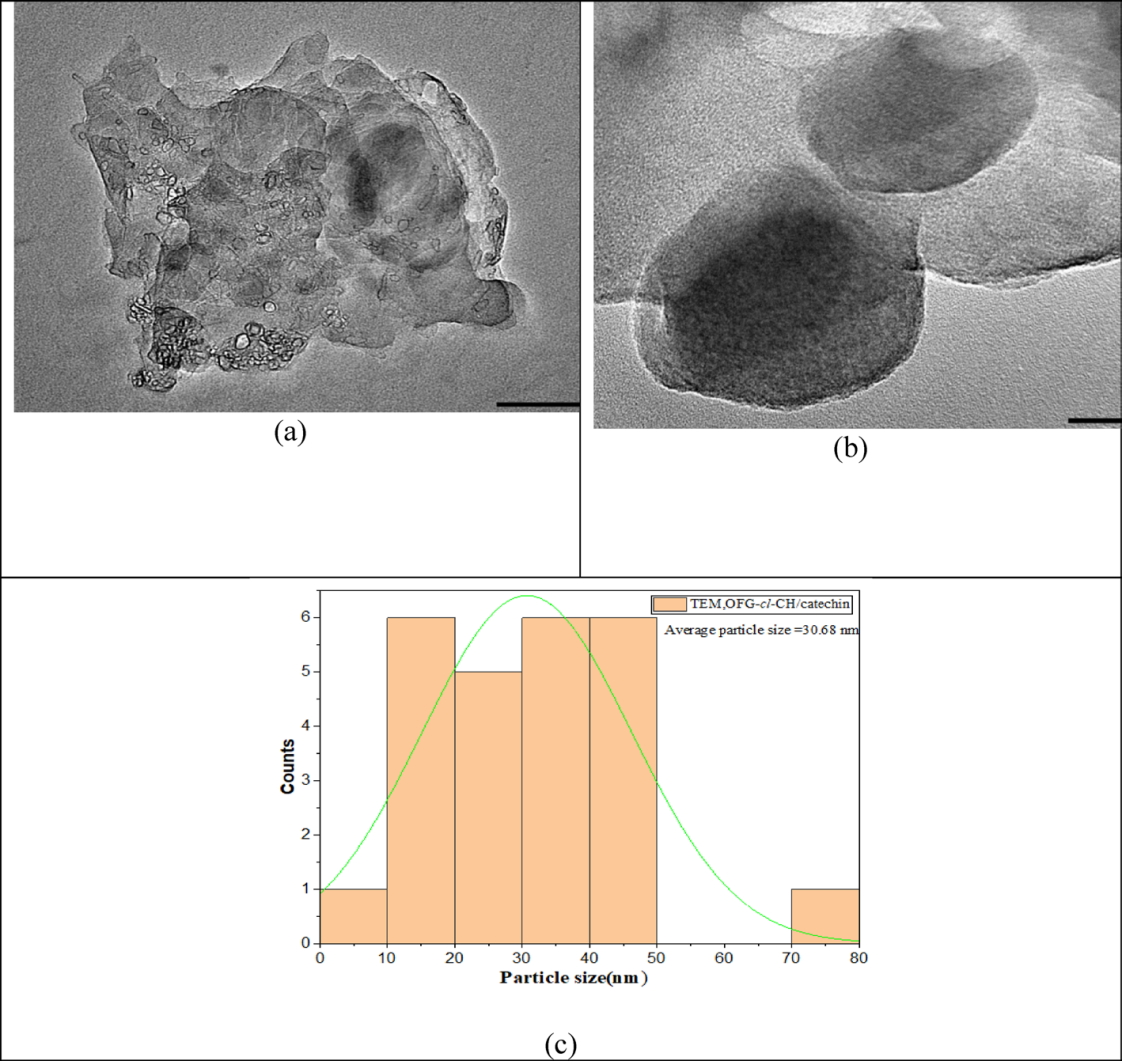
TEM image of OFCC nanocomposite gel matrix** a** at 100 nm scale);** b** at (20 nm scale);** c** Catechin particle size distribution

**Fig. 12:**
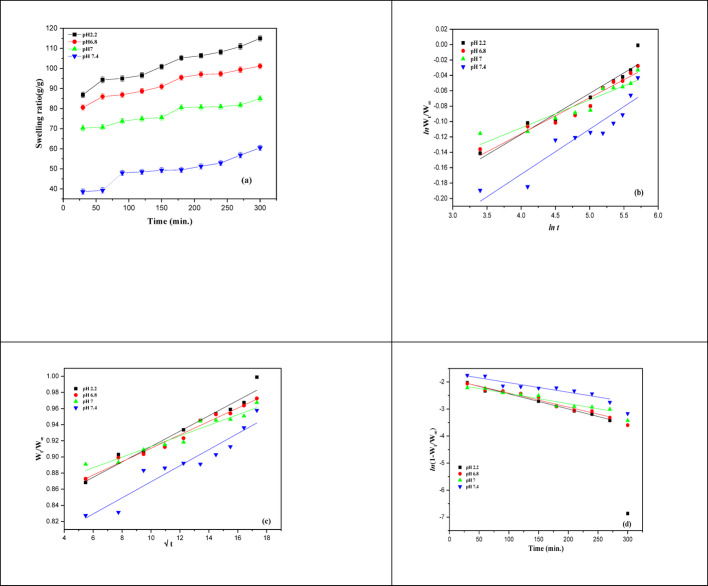
**a**–**d**: (**a**) Swelling profile of OFCC nanocomposite gel matrix(catechin 2500 µg/ml, encapsulated by in-situ method); (**b**)Plot of *ln*W_t_/W_∞_ versus *ln t*; (**c**) Plot of W_t_/W_∞_ versus √ t; (**d**) Plot of *ln*(1-W_t_/W_∞_) versus Time, for OFCCnanocompositegel matrix as a function of pH

Swelling behavior of hydrogels is a vital property which depends upon the chemical architecture of the gel network and thus decides their end use. In present study, swelling kinetics of OFCC nanocomposite gel matrix had been evaluated as a function of pH (Fig. 11 (a–d)); (Supplementary Fig. S8(a–d) & Fig. S9(a-d)) and ionic strength of swelling mediums (Fig. 12 (a–d))at 37 °C.OFCC gel matrix synthesized through in situ method had depictedhigher swelling compared to matrix fabricated through equilibrium method. With rise in concentration of catechin, swelling declined which might be due to formation of more compact three-dimensional networks [61]. OFCC matrix represents relatively less swelling compared to OFC. This behaviour clearly indicates that incorporation of catechin nanoparticles in the gel matrix facilitate more ionic interactions thus making the gel matrix more compact.

With respect to pH of swelling medium, OFCC matrix depictedhigher swelling profile in acidicpH. This behaviour was due to protonation of free NH_2_of chitosan and -C = NH groups on polymeric matrix. Protonation of positively charged moieties causes repulsion and hence were responsible for higher swelling in acidic pH [59]. Swelling of OFCC matrix was drastically less in 0.9% NaCl than in DDW which was due to phenomenon of ex-osmosis which caused swollen gel to shrink in the presence of electrolytes. After 24 h equilibrium swelling the gel matrix synthesized through in situ and swelling equilibrium method had shown 51.28% and 34.44% swelling respectively. Swelling kinetics and diffusion mechanism of the OFCC gel matrix had also been evaluated using Ritger & Peppas equations. The aforementioned matrices’ diffusion constant (n) and gel characteristic constant (K) had been analyzed from plot of *ln* W_t_/W_∞_ versus *lnt* (Figs. 11(b), 11(b), S8(b) and S9(b). Value of n and K had been reported in the Table 1. As value of ‘n’ was smaller than 0.5 in all cases which represents pseudo-Fickian diffusion as prominent swelling mechanismin present case. Inpseudo-Fickian diffusion mechanism, curves resemble Fickian curves but approaches equilibrium at a slower pace. There is no characteristic boundaries between swollen and dry region in polymeric matrix. Higher crosslink density reduces the free volume in the hydrogel networks, which slows solvent penetration and limits polymer chain relaxation, leading to pseudo Fickian diffusion behavior. On the other hand, lower cross-linking density increases network flexibility, allowing faster polymer relaxation relative to solvent diffusion.

**Table 1:** Results of diffusion exponent (*n*) and gel characteristic constant (K) for swelling kinetics of OFCC nanocomposite gel matrix (2500 µg/ml &5000 µg/ml catechin encapsulated by in-situ method and catechin 2500 µg/ml, encapsulated by Swelling equilibrium method) in different swelling medium at 37 °C

		OFG-cl-CH-catechin (Catechin 2500 µg/ml: in-situ method)		OFG-cl-CH-catechin (Catechin 5000 µg/ml: in-situ method)		OFG-cl-CH-catechin (Catechin 2500 µg/ml: swelling equilibrium method	
S.no.	Swelling parameter	Diffusion exponent (*n*)	Gel characteristic constant K× 10	Diffusion exponent (*n*)	Gel characteristic constant K× 10	Diffusion exponent (*n*)	Gel characteristic constant K× 10
1	pH 2.2	0.05327	7.19	0.01685	8.83	0.08042	6.03
2	pH 6.8	0.04686	7.38	0.02373	8.08	0.05031	7.24
3	pH 7	0.03642	7.76	0.03454	7.28	0.07073	6.37
4	pH 7.4	0.05865	6.68	0.04709	7.55	0.04344	7.78
5	0.9% NaCl	0.04996	7.90	0.0483	8.44	0.09314	6.73

Swelling diffusion coefficients are used to predict the pace at which the swelling media will penetrate the gel matrix. Plot of √t vs. *Wt/W ∞* was used toevaluate initial diffusion coefficient (Di) and average diffusion coefficient (D_A_). D_i_ and D_A_ as a function of pH and ionic strength of swelling media have been evaluated using Eqs. (6) and (7) (Fig. 11(c) and Fig. 12(c) (Supplementary Figs. S8(c) & S9(c)). Late diffusion coefficient (D_L_)of each gel matrix was evaluated by plotting graph *ln*(1-W_t_/W_∞_) vs. ‘t’ as shown in Fig. 11(d), Fig. 12(d); (Supplementary Figs. S8(d) and S9(d). Results for D_i_, D_A_and D_L_had been presented in Table 2. In all swelling media, Di was found to be less than D_L_ with the exception of 0.9% NaCl solution. This behavior was due to the phenomenon of slow penetration of swelling media at initial phase due torestricted chain relaxation and compact/cross-linked networks. At later stage of swelling, simultaneous chain relaxation facilitated increased swelling. In present work, OFCC gel encapsulated with 2500 µg/ml catechin exhibited optimal swelling and pH responsive behaviour thereby making it suitable candidate for drug release studies since eequilibrium swelling ratio (ESR) of hydrogels exerts an influence on their drug release rates [39]. ESR of OFCC nanocomposite was found to be 115.20 ± 1.42%, 106 ± 1.42%, 91.19 ± 1.23% and 67.49 ± 1.46% at pH2.2, pH6.8, pH7and pH7.4 respectively.

### Drug release study of MTX through OFC and OFCC gel matrix

#### Loading and in vitro release dynamics of MTX from OFC-MTX and OFCC-MTX hydrogels in different release mediums

MTX was loaded into the OFC and OFCC functionalized gel matrix using the swelling equilibrium method. Drug’s entrapment efficiency had been evaluated using Eq. 9. After 24 h, up to 55.68% and 49.56% of entrapment efficiency was achieved in OFCand OFCC nanocomposite hydrogel respectively. Lower entrapment efficacy (%) acquired by OFCC was might be due to formation of tightly cross-linked networks owing to embedded catechin nanoparticles, which decreases the accessible space in the hydrogel matrix.

**Fig. 13:**
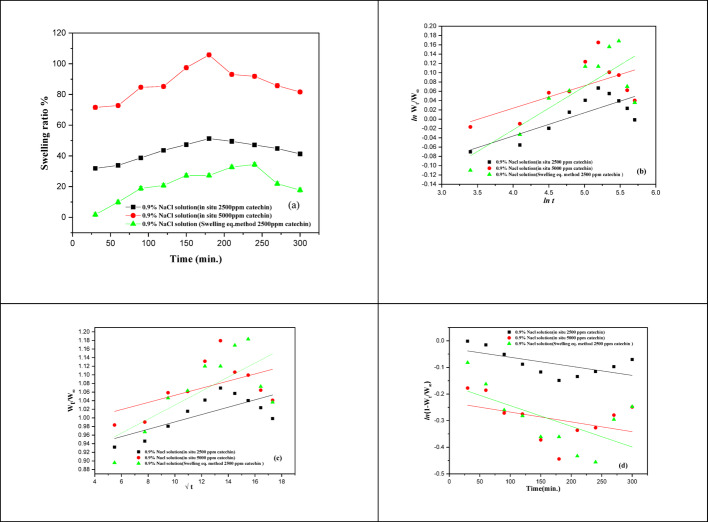
**a**–**d**: (**a**)Swelling profile of OFCC nanocomposite gel matrix (catechin 2500 µg/ml&5000 µg/ml, encapsulated by *insitu* method and catechin 2500 µg/ml, encapsulated by swelling equilibrium method, ); (**b**) Plot of *ln*W_t_/W_∞_ versus *ln* t; (**c**) Plot of W_t_/W_∞_ versus √ t; (**d**) Plot of *ln*(1-W_t_/W_∞_ ) versus Time, for OFCC nanocomposite gel matrixas a function of 0.9% NaCl solution

In vitro release dynamics of the MTX from the drug loaded OFC and OFCC gel matrix had been evaluated in different release mediums with variable pH viz.: pH2.2, pH7, pH7.4, SGF of pH 1.2and SIFof pH6.8. Results of drug release were shown in Fig. S10(a–b) & Fig. 13 (a-b). It had been observed that amount of drug released (µg/ml) from the per gram of the gel was higher in SGF (pH1.2) and in acidic pH 2.2 than in SIF (pH6.8), pH7 and pH7.4. Two factors might be responsible for such behaviour: (i) higher solubility of MTX in lower pH; (ii) presence of amino/imine groups in OFC and OFCC hydrogels which had shown higher swelling at low pH (acidic medium). Therefore, drug release and swelling profile of functionalized gel matrixhad shown clear correlation as confirmed by the swelling and drug release results (Fig. S13(a, b)). Zheng and colleagues have reported similar results where MTX was released more readily from drug-loaded ganoderma lucidum polysaccharide hydrogels in an acidic solution [62].

Cumulative percentage of drug release from gel matrix as a function of pH was calculated using Eq. 10 and results are presented in Fig. S10(b) &Figure 13(b).In OFC-MTX hydrogel matrix, maximum cumulative release (83.78%) was achieved in SGF (pH 1.2), and minimum in pH 7.4 (31.05%) after 24 h. In OFCC-MTX gel matrix, cumulative release upto 75.19% and 23.98% were achieved in SGF (pH 1.2) and pH 7.4 respectively after 24 h. Amount of the drug released per gram of the gel was higher in SGF than in SIF for both the hydrogels. Lower cumulative % release in pH 7.4 was might be due to lower swelling ability of both matrix in higher pH. These results were found well correlated with the swelling behavior of gel since there was strong interactions between the functional groups present on the polymeric matrix and MTX molecules as depicted from FTIR spectra. These results were found consistent with release profile study of MTX from curcumin-based PEG nanocomposite hydrogels which had also demonstrated that MTX could be stable at normal pH of physiological fluid (neutral medium) and release rate of MTX increased as pH of the physiological fluid decreased [57].Under acidic pH, the ionization of MTX molecules’ amino groups and the imine bond in the gel matrix led to increased repulsive forces, resulting in increased chain relaxation and drug release. As a result, current findings suggest the applicability of OFC and OFCC gel matrix for the controlled drug delivery of MTX in acidic environment [57].The time required for 50% of total release of MTX molecules in SGF was 860 minutesfrom OFC-MTXand 958 min from OFCC-MTX hydrogels suggesting faster release from OFC-MTX hydrogel which was consistent with the higher drug loading by OFC-MTX matrix as well (Fig. 13(b) and Fig. S10(b)). There was no initial burst release of drug from both polymeric matrices.

#### Drug release kinetics of MTX from OFC-MTX and OFCC-MTX gel matrix in different release mediums

**Fig. 14:**
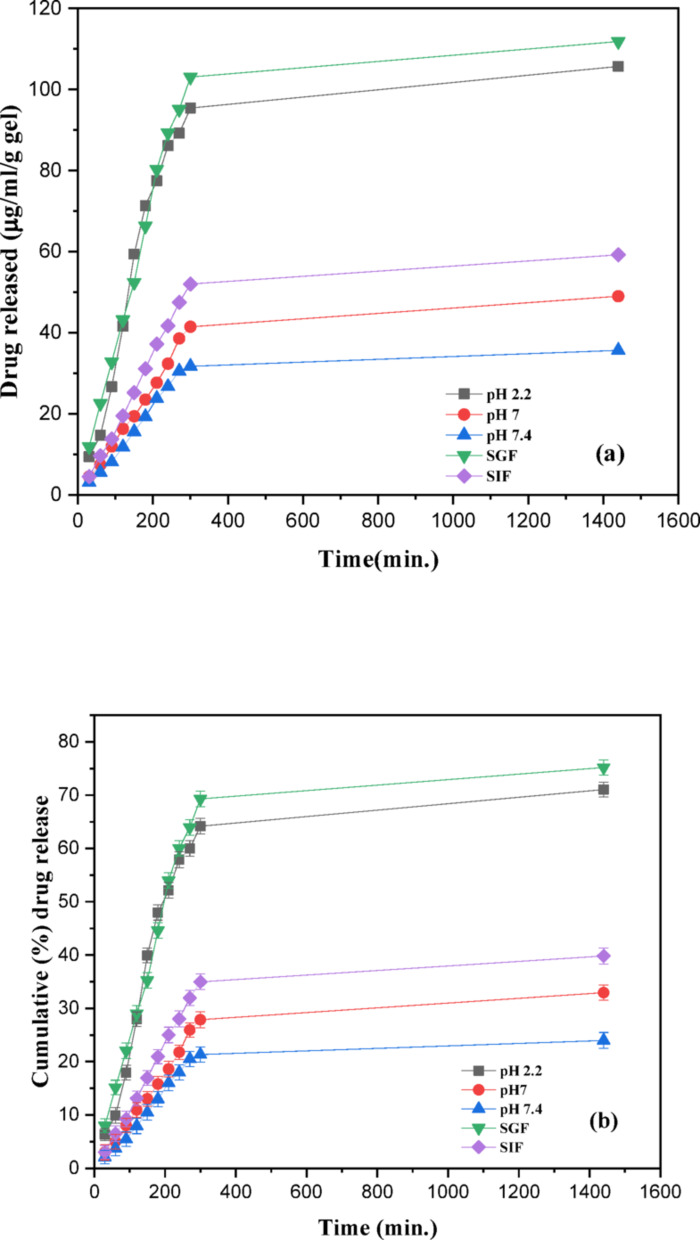
**a**–**b**: (**a**) In vitro release dynamics of MTX from drug loaded OFCC-MTX nanocompositegel matrix in different release mediums at 37 °C; (**b**) Cumulative (%) of total drug release from drug loaded OFCC-MTX nanocomposite gel matrixin different release mediums at 37 °C

Drug release kinetics is useful technique utilized for predicting the mechanism of drug release. In present study, in vitro release profile of MTX from a drug-loaded polymeric matrix had been analyzed using different kinetic models viz.: zero order, first order, Higuchi model, and Korsmeyer-Peppas model. The plot of zero order, first order, Higuchi model and Korsmeyer–Peppas kinetics models at different release mediums with variable pH had been evaluated using Eqs. (11, 12, 13 & 14) respectively and results were presented in Fig. 14(a–d) (Supplementary Fig. S11(a–d); Table 3. The regression coefficient (R^2^) value close to unity had been used to identify the best fitted model for the release kinetics. During drug release, Korsmeyer–Peppas kinetics model was found best fitted with highest R^2^ value for both matrix (OFC and OFCC) in all release mediums.

**Table 2:** Results of diffusion coefficients D_i_, D_A_ and D_L_for swelling kinetics of OFCC nanocomposite gel matrix (2500 µg/ml & 5000 µg/ml catechin encapsulated by in-situ method and catechin 2500 µg/ml, encapsulated by Swelling equilibrium method) in different swelling medium at 37 °C

		OFG-cl-CH-catechin (Catechin 2500 µg/ml: in-situ method)			OFG-cl-CH-catechin (Catechin 5000 µg/ml: in-situ method)			OFG-cl-CH-catechin (Catechin 2500 µg/ml: Swelling equilibrium method			
S.no.	Swelling parameter	D_i_ × 10^5^	D_A_×10^3^	D_L_ ×10^5^	D_i_ × 10 ^5^	D_A_×10^3^	D_L_×10^5^	D_i_ × 10 ^5^	D_A_×10^3^	D_L_×10^5^	
1	p pH 2.2	1.48	1.96	7.06	0.301	1.84	8.90	2.63	1.20	7.69	
2	pH6.8	1.33	1.88	6.54	0.378	1.74	2.60	1.42	1.03	9.20	
3	pH7	0.76	1.74	4.72	0.902	1.71	2.90	2.57	0.96	7.62	
4	pH7.4	1.64	1.50	4.48	1.55	1.49	15.78	0.942	0.69	15.63	
5	0.9% NaCl	1.43	1.17	0.43	1.34	1.57	0.46	5.19	0.67	0.97	

**Fig. 15:**
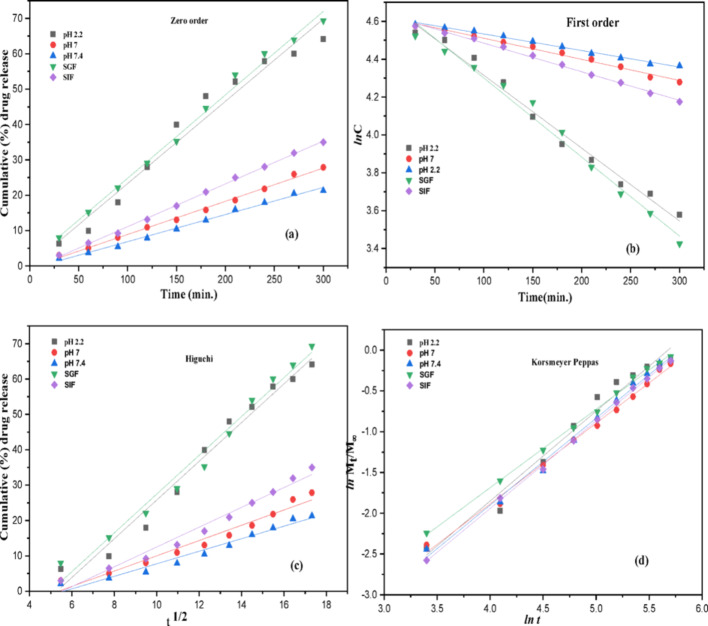
**a**–**d**: (**a**) Plot of zero order Kinetic model ; (**b**) Plot of First order Kinetic model; (**c**) Plot of Higuchi model; (**d**) Plot of Korsmeyer-Peppas kinetic model for in vitro release dynamics of MTX from drug loaded OFCC-MTX nanocomposite gel matrix in different release mediums at 37 °C

**Fig. 16:**
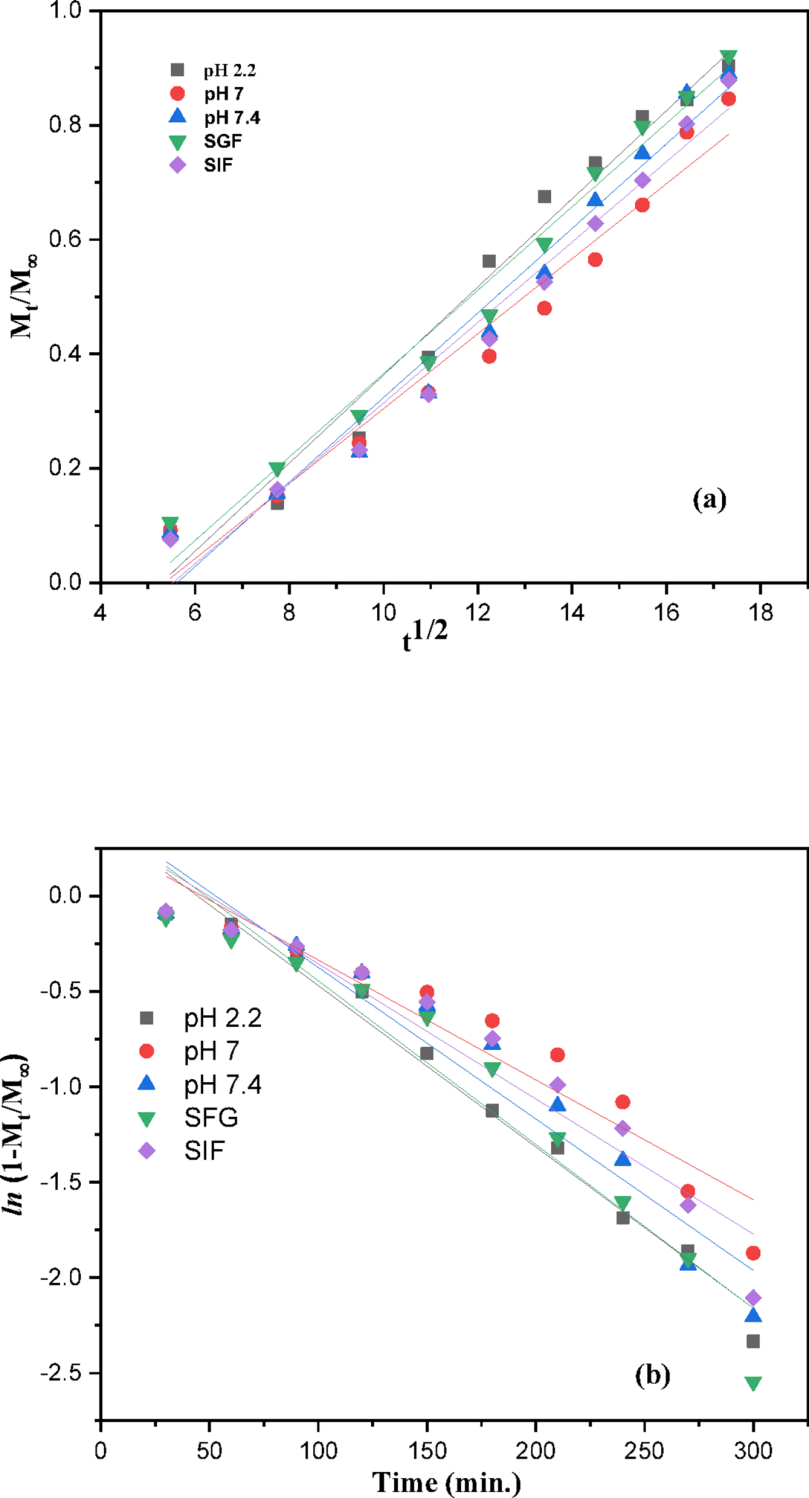
**a**–**b**: (**a**) Plot of M_t_/M_∞_versus t^1/2^ to calculateD_i_& D_A_; (**b**) Plot of *ln*(1-M_t_ /M_∞_) versus Time to calculate D_L_for MTX release fromdrug loaded OFCC-MTXnanocomposite gel matrix in different release mediums at 37 °C

To predict the drug release mechanism, diffusion exponent (n) and gel characteristic constant (‘K’) had been evaluated using Eq. (15) (Fig. S11(d), Fig. 14(d); Table 4). InOFC-MTX matrix, drug release had followed non-Fickian diffusion mechanism in all release mediums with an exception in SIF where case II diffusion mechanism was prominent one. In case of OFCC-MTX nanocomposite gel matrix, Case II diffusion was predominant mechanism. Case II diffusion occurs when polymeric chain relaxation process is predominant whereas drug diffusion and polymeric chain relaxation rates are comparable in Non–Fickian diffusion mechanism. D_i_, D_A_ andD_L_as a function of pH during drug release hadalso been calculated using Eqs. (16), (17) & (18) respectively (Supplementary Fig. S12(a-b), Fig. 15(a–b); Figure 16(a-b),Table 4). In SGF, the value of D_i_was observed to be more than the values of D_L_which had shown that in initial period of drug release, diffusion of drug from gel matrix was faster than in later stages. At later stages, chain relaxation becoming controlling factor for drug release.

**Table 3:** Drug Release parameters obtained from different kinetic models for MTX release from OFC-MTX and OFCC-MTX matrix in different release mediums at 37 °C Drug Release parameters obtained from different kinetic models for MTX release from OFC-MTX and OFCC-MTX matrix in different release mediums at 37 °C

Drug release gel matrix →		OFC-MTX					OFCC-MTX				
Kinetic model	Parameter	pH 2.2	pH 7.0	pH 7.4	SGF	SIF	pH 2.2	pH 7.0	pH 7.4	SGF	SIF
Zero order	R^2^	0.9997	0.9995	0.9960	0.9950	0.9988	0.9657	0.9956	0.9937	0.9936	0.9988
	K^0^_**0**_	0.2320	0.1248	0.0932	0.2538	0.1364	0.2326	0.0939	0.0767	0.2359	0.1210
First order	R^2^	0.9699	0.9973	0.9970	0.9759	0.9973	0.9865	0.9897	0.9926	0.9854	0.9956
	K_1_	0.0041	0.0016	0.0011	0.0053	0.0018	0.0039	0.0011	0.0009	0.0042	0.0015
Higuchi	R^2^	0.9746	0.9796	0.9813	0.9848	0.9813	0.9755	0.9614	0.9668	0.9793	0.9738
	k_H_	5.3633	2.8926	2.1657	5.9100	3.1665	5.4741	2.1598	1.7718	5.4833	2.7994
Korsmeyer-Peppas	R^2^	0.9997	0.9986	0.9963	0.9961	0.9976	0.9768	0.9948	0.9938	0.9978	0.9990
	n	0.987	0.893	0.878	0.835	1.114	1.102	0.989	1.066	0.961	1.074

**Table 4:** Results of diffusion exponent 'n' and gel characteristic constant 'k' and various diffusion coefficients for the release of MTX from OFC-MTX and OFCC-MTX in different release mediums at 37 °C

Release medium	Diffusion exponent, ‘*n*’	Gel characteristic constant, ‘k’×10^3^	Diffusion coefficients (cm^2^/min)		
			Initial	Average	Late
			Di×10^5^	D_A_×10^5^	D_L_×10^6^
*OFC-MTX*					
Distilled water	0.893	5.42	2.0	2.70	2.08
pH 2.2 buffer	0.987	3.20	2.11	3.62	2.14
pH 7.4 buffer	0.878	6.16	2.15	2.29	2.34
SGF	0.835	8.06	2.19	23.77	2.68
SIF	1.114	1.61	2.22	.75	2.02
*OFCC-MTX*					
Distilled water	0.989	2.91	2.62	3.46	2.41
pH 2.2 buffer	1.102	1.91	1.90	2.36	1.79
pH 7.4 buffer	1.066	2.10	2.41	2.01	2.26
SGF	0.961	3.96	2.34	3.56	2.45
SIF	1.074	1.96	2.18	2.59	2.02

Additionally, comparative account of cumulative drug released (%) by OFC-MTX and OFCC-MTX hydrogels with other functionalized gels have also been conducted (Table 5 and 6). It has been observed that in SGF, cumulative drug release % was higher from OFC-MTX hydrogel (83.78%) than OFCC-MTX (75.19%). Present study was focused on preliminary synthesis, characterization and biological validation of functionalized fenugreek gum-chitosan hydrogels under in vitro conditions only. However, cytotoxicity, cell viability/in vivo studies are further required to bring this matrix at clinical plate form. This is the limitation of the present study.

### Biomedical properties of polymers

The biodegradability and biocompatibility of polymeric hydrogels under physiological conditions are two key features that can be used to determine their biomedical usage. Thrombogenicity and haemolytic potential of FG, OFG, OFC and OFCC hydrogel samples were studied as per the procedure mentioned in Sect. 2.8.1 and 2.8.2. After treating 2 ml of ACD blood with native gum, OFG and functionalized gel matrix, it was found that clot and thrombus development had occurred. Weight of the respective blood clot formed and thrombose % were presented in Table 7. Both gel matrix (OFC and OFCC) exhibited non-thrombogenic behaviour, as evidenced by the 86.7% and 78.9% thrombus formation respectively. Weight of the clots generated was up to (0.112 ± 0.0018), (0.108 ± 0.0011), (0.111 ± 0.0023), and (0.101 ± 0.0015) forFG, OFG, OFC and OFCC nanocomposite thus demonstrateing biocompatible behaviour of these gels. Haemolytic index values achieved upto 4.03%, 3.64%, 3.41%, and 2.61% for FG, OFG, OFC and OFCC respectively. It has already been documented that materials having haemolytic potential of less than 5% have been demonstrated to be biocompatible and become appropriate for use in medical applications [57]. According to ASTM, materials can be classified into three different categories according to their hemolytic index (hemolysis %). Materials which have haemolysis % more than 5% are classified as hemolytic, while 2–5% is classified as slightly hemolytic and less than 2% are considered as non-hemolytic materials [51]. The observed slightly hemolytic behavior of the synthesized OFCC hydrogel may be attributed to its favorable physicochemical characteristics, including nanoparticle size, smooth surface morphology and near-neutral /slightly negative charge. Due to these properties, electrostatic interaction and mechanical disruption of the erythrocyte membrane is minimum. Furthermore, catechin embedded hydrogel components help in maintain membrane integrity and thereby preventing hemolysis.

**Table 5:** Comparative account of OFG-cl-CH and OFG-cl-CH-catechin nanocomposite hydrogel with other hydrogel matrix for in vitro drug release

Hydrogel matrix	Drug	Cumulative drug release % (CDR%) medium	References
Dialdehyde konjac glucomannan /chitosan Schiff’s base	Ofloxacin	62.5/pH 2.2	[51]
Chitosan/dialdehyde xanthan gum interpenetrated withhydroxypropyl methylcellulose(HC)(CXH hydrogel)	Ampicillin trihydrate(AT) Rifampicin (RC) Minocycline hydrochloride	≥ 50 /SGF ≥ 50 /SGF ≥ 50 /SGF	[63]
N,O-carboxy methyl chitosan/multialdehyde guar gum Schiff base	Doxorubicin	≥50 /SGF	[64]
Alginate-gelatin Alginate-gelatin/Fe3O4	Doxorubicin hydrochloride(DOX)	~ 42/pH 4 ~ 42/pH 4	[65]
Dextran- ethylenediamine/Fe3O4	Doxorubicin hydrochloride(DOX)	45.6/pH 5.5	[66]
OFG-cl-CH Schiff base	Methotrexate(MTX)	83.78/SGF	Present work
OFG-cl-CH-catechin Nanocomposite hydrogel	Methotrexate(MTX)	75.19/SGF	Present work

**Table 6:** Comparative account OFCC-MTX and OFCC-MTX nanocomposite hydrogel with other hydrogel matrix for invitro MTX drug release

Hydrogel matrix	Methods Of formation	Drug	Cumulative drug release % (CDR%) medium	References
Magnetic K-carrageenan/chitosan hydrogel	In situ	MTX	68% blood pH 7.4	[67]
MTX/AEM-CS hydrogel AEM-: Abelmoschus esculentus mucilage CS-: Chitosan	Coacervation method	MTX	53.17% tumor cell pH5.5	[68]
APDMS crosslinked CG/SA/PVA hydrogel APDM-: 3-aminopropyl(diethoxy)methyl Silane CG-: Carrageenan SA-: Sodium alginate PVA-: Polyvinyl alcohol	Solution casting	MTX	81.25% blood pH 7.4	[69]
OFC-MTX	Oxidative route Insitu gelation & self-crosslinking method	MTX	83.78% SGF pH1.2	Present work
OFCC-MTX	Oxidative route *Insitu* gelation & self-crosslinking method	MTX	75.19% SGF pH1.2	Present work

The heartwood of the *Acacia catechu* tree is said to contain a high level of antioxidant activity and to be a great source of flavonoids, with epicatechin and catechin [27]. Fenugreek gum and chitosan, base backbone component of the current matrix also possess antioxidant properties, since they had flavonoids and polyphenols, as well as free amino groups, respectively. Consequently, it is necessary to examine the antioxidant potential of parent backbone and functionalized gel matrix. Antioxidant activity of catechin, OFC, OFCC (2500 µg/ml) and OFCC(5000 µg/ml) had been evaluated by the DPPH scavenging assay. The assay for DPPH scavenging revealed scavenging percentages of 77 ± 1.73%, 55 ± 1.52%, 57.53 ± 1.01%, and 58.41 ± 0.83% for catechin, OFC, OFCC (2500 µg/ml) and OFCC(5000 µg/ml)respectively (Table 8). The addition of catechin to the gel matrix increased free radical scavenging ability of functionalized nanocomposite. Additionally, there was slight increase in antioxidant behaviour, from 57.53% to 58.04%, with rise in concentration of catechin. Additionally, visual analysis revealed that the DPPH scavenging assay changed from purple to yellow. During study of antioxidant behaviour, these samples caused the [2, 2’-diphenyl-1-picrylhydrazyl] radical to be abstracted, resulting in 1,1-diphenyl-2picrylhydrazine formation (Fig. 3(a-b).Catechin act as a natural antioxidant, polyphenolic cross-linking and reinforcing agent in the OFC matrix. Due to multiple hydroxyl groups present on the catechin structure, it forms strong hydrogen bonding and secondary interaction with the imine (-C = N-) linkages, thereby enhancing the mechanical strength and structural stability of the Schiff’s base. Catechin primarily act as a multifunctional bionanoparticle, contributing antioxidant activity, acting simultaneously as a natural polyphenolic structurally compatible functional component within the hydrogel that enhances structural integrity and thermal stability that contributes to the system biomedical performance.

**Table 7:** Results of antioxidant behaviour of catechin, OFG, OFC and OFCC gel matrix

Sample/Gel matrix	Weight of blood clot (g)	Thrombose (%)	Inference	Blood compatibility
*Thrombogenicity*				
FG	0.112 ± 0.0018	87.50	Non-thrombogenic	Blood compatible
OFG	0.108 ± 0.0011	84.37	Non-thrombogenic	Blood compatible
OFC	0.111 ± 0.0023	86.71	Non-thrombogenic	Blood compatible
OFCC	0.101 ± 0.0015	78.90	Non-thrombogenic	Blood compatible
*Haemolytic index*				
FG	0.270 ± 0.0029	4.03	Slightly-haemolytic	Blood compatible
OFG	0.260 ± 0.0025	3.64	Slightly-haemolytic	Blood compatible
OFC	0.254 ± 0.0017	3.41	Slightly-haemolytic	Blood compatible
OFCC	0.233 ± 0.0029	2.61	Slightly-haemolytic	Blood compatible

### In vitro hydrolytic degradations studies

**Fig. 17:**
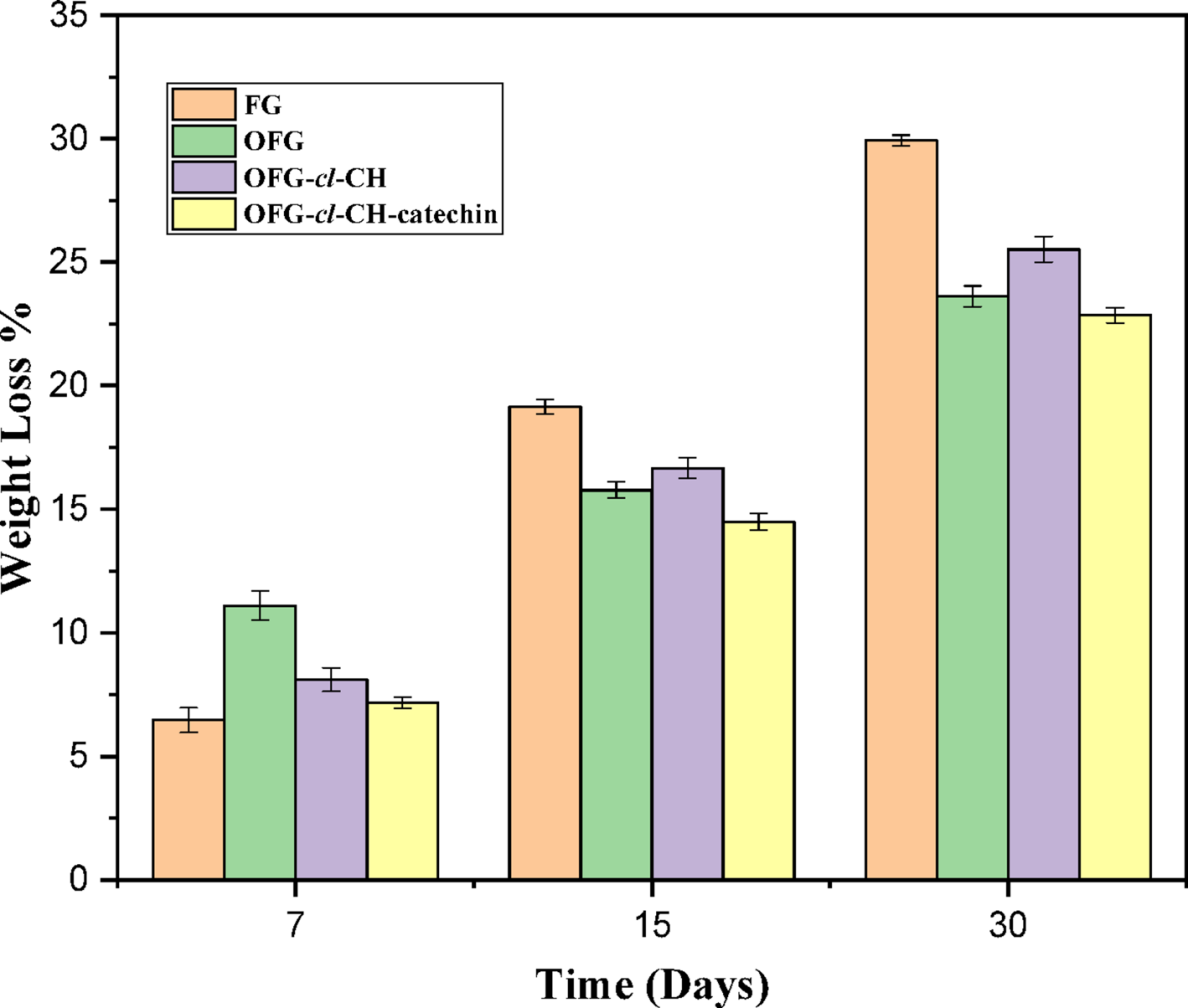
Hydrolytic degradationbehaviour of FG, OFG, OFC gel matrixand OFCC nanocomposite gel matrix in PBS of pH7.4 at 37 °C

In present work, biodegradation potential of parent and functionalized gels were evaluated through in vitro hydrolytic degradation at physiological pH conditions (pH 7.4; 37℃) for 30 days and results were shown in Fig. 17; Table 9. It was observed that initially there weight gain by hydrogels which was due to swelling of gel and after attaining equilibrium swelling, a consecutive weight loss was observed. Weight loss (%) was recorded after every week (Table 9). Native gum FG showed highest weight loss % among all samples. OFG decomposed slowly than native FG. According to literature survey, oxidized xanthan gum degraded less quickly than xanthan gum when it was cross-linked with gelatin, which had suggested that oxidation with sodium periodate enhanced the gum’s natural resilience [36]. Similarly in Schiff base matrix (OFC), there was formation of imine bond between carbonyl groups of OFG andNH_2_ groups of chitosan-through compatible self-crosslinking mechanism. Cross-linking resulted in decline in degradation percentage of OFC as compared to native gum. Similarly, incorporation of catechin further decreased degradation (%) and improves gel stability [70]. Similar results had been reported in literature. Incorporation of ZnO nanoparticles in alginate aldehyde/gelatin–ZnO nanocomposite decreased the degradation % of functionalized gel matrix as compared to the alginate aldehyde /gelatin hydrogels networks [71–74]. Therefore, in present work, catechin based nanocomposite hydrogel networks possess improved stability and durability. Amalgamation of biodegradable natural polymers, ideal biocompatible potential, and excellent water holding behaviour of OFC and OFCC, make this gel matrixas suitable candidate for in vitrodrug release study.

**Table 8:** Results of hydrolytic degradation of FG, OFG, OFC and OFCC gel matrix at 37 °C

Sample/ Gel matrix	DPPH radical scavenging (%)	Inference
Catechin	77 ± 1.73	Antioxidant
OFC	51 ± 1.52	Antioxidant
OFCC containing (2500 µg/ml catechin)	57.53 ± 1.01	Antioxidant
OFCC containing (2500 µg/ml catechin)	58.41 ± 0.83	Antioxidant

**Table 9:** Results of hydrolytic degradation of FG,OFG, OFC and OFCC gel matrix at 37 °C

Weight loss (%)				
DAYS	FG	OFG	OFC	OFCC
**7**	6.47 ± 0.50	11.11 ± 0.58	8.11 ± 0.48	7.17 ± 0.23
**15**	19.15 ± 0.30	15.77 ± 0.33	16.66 ± 0.41	14.49 ± 0.33
**30**	29.93 ± 0.22	23.62 ± 0.42	25.52 ± 0.52	22.85 ± 0.31

## Conclusion

In the present study, a novel catechin embedded oxidized fenugreek gum/chitosan-basedpH responsive nanocomposite gel matrix were fabricated through green oxidative route using sodium periodate as oxidant via self-cross-linking method. Further in vitrorelease profile of an anticancer drug MTX had been evaluated from OFC and OFCC gel matrix. Physico-chemical evaluated of catechin and structural characterization of gel matrix had been carried out via FTIR, powdered XRD, FESEM, TEM, TGA, LC/MS and HPLC analysis. XRD results had confirmed particle size of catechin nanoparticles upto 31.04 nm and TEM analysis confirmed their spherical shapes. Swelling kinetics of functionalized gels was analyzed as a function of pH and ionic strength of swelling medium. Swelling of OFCC nanocomposite polymeric hydrogel was occurred through pseudo-Fickian diffusion mechanism. In vitro release behaviour of MTX from drug loaded matrix (OFC-MTX and OFCC-MTX) had also beenevaluated in different release mediums with reference to zero order, first order, Higuchi model and Korsmeyer–Peppas kinetics models. MTX release was achieved higher in pH 2.2 and SGF as compared to pH7.4 and in SIF. Case-II diffusion mechanism was prominent one for drug release from bothOFC and OFCC hydrogels which was mechanistically decoupledto swelling mechanism (pseudo-Fickian), as swelling primarily reflects solvent diffusion into the hydrogel matrix, whereas drug release is additionally governed by polymer relaxation and matrix reorganization. Drug release mechanism was consistent with Korsemeyer-Peppas model for both gel matrix. In early stages, the rate of drug release was found higher than the later stages thereby favored a controlled manner drug release pattern after attaining equilibrium. OFCC nanocomposite gel matrix had shown biocompatible and non-thrombogenic behavior along with antioxidant behavior. Since free radicals are also generated in cancerous tissues, therefore present matrix could further be screened for in vivo screening at cell line level as well as in vivodrug release of anticancer drugs in future. Present study provided an experimental basis for the green route synthesis of OFG-cl-CH-catechin (OFCC) nanocomposite hydrogelsas potentially suitable drug delivery device for protecting and delivering MTX anticancer drug to improve its bioavailability. Hence, the findings of the present study may be useful in controlled drug delivery applications (in vitro) due to incorporated antioxidant behaviour and biodegradable characteristic. Biomedical suitability of present matrix was examined through in vitro analysis. Further in vivo analysis study is required for making the matrix applicable for clinical applications.

### Limitation of the study

Although present gel matrix provide a structural and synthetic roadmap for polyphenol integrated self cross-linked gel matrix useful for in vitro drug release study with supported experimental and characterization results but there are certain limitations such as mechanical/tensile strength evaluation, in vivo cytotoxicity/cytocompatibility and in vivo drug release behaviour have not been studied. Therefore these studies have to evaluate further before applying it for real time clinical platform.

## Supplementary Information

Below is the link to the electronic supplementary material.

Supplementary Material 1.

## Data Availability

All data generated or analyzed during this study are included in this published article.
