# Supplementary material for: Synthesis, characterization and in vitro release kinetics of methotrexate from oxidized fenugreek gum/chitosan-catechin bionanocomposite hydrogel matrix
Source: Discov Nano. 2026 Jun 24;21(1):283. doi: 10.1186/s11671-026-04698-0 (PMC13294432; doi:10.1186/s11671-026-04698-0)
Supplement: Supplementary file 1 — Supplementary Material 1. [file 11671_2026_4698_MOESM1_ESM.docx]

Synthesis, Characterization and *In Vitro* Release Kinetics of Methotrexate from Oxidized fenugreek gum/chitosan-catechin bionanocomposite hydrogel matrix

**Ganesh Kumar^1^, Nisha Sharma^1*^and Younis Ahmad Hajam^2*^**

**
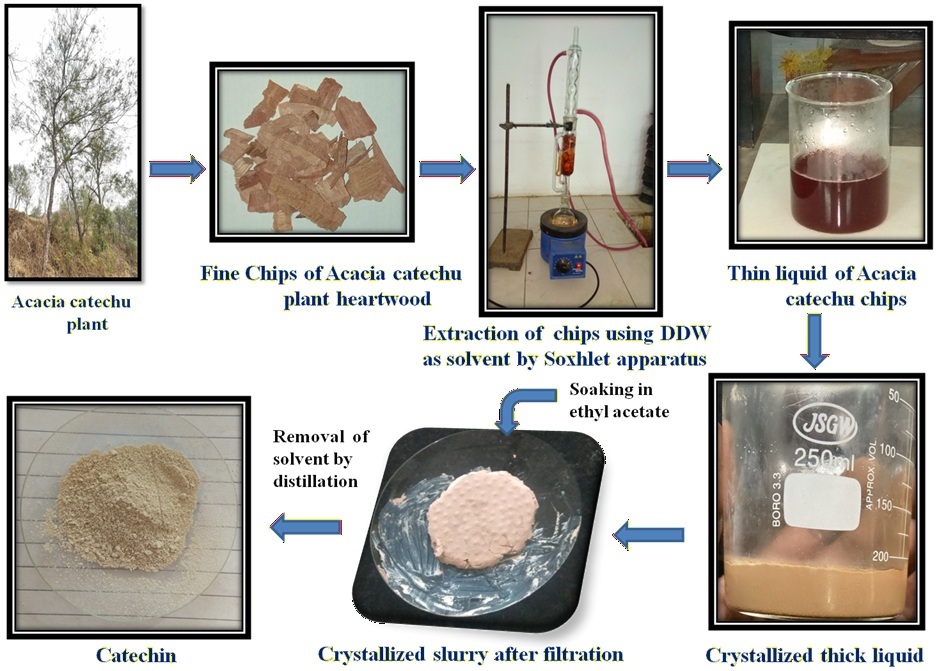
**

**Fig.S1: (a-b) Pictorial representation of stepwise extraction process of catechin from *Acacia catechu* heartwood**

| **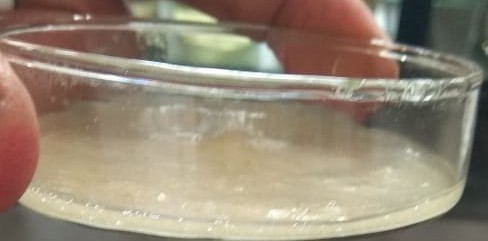OFG-*cl*-CH Schiff’s base**  **(a)**  **hydrogel** | **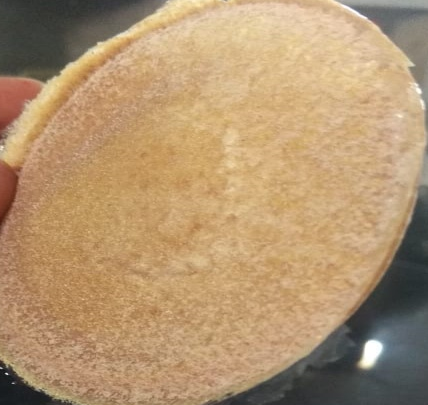OFG-*cl*-CH Schiff’s base network**  **(b)** |
| --- | --- |
| **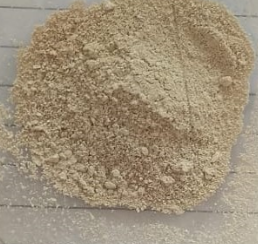Catechin extracted from**  **(c)**  ***Acacia catechu* plant wood** | **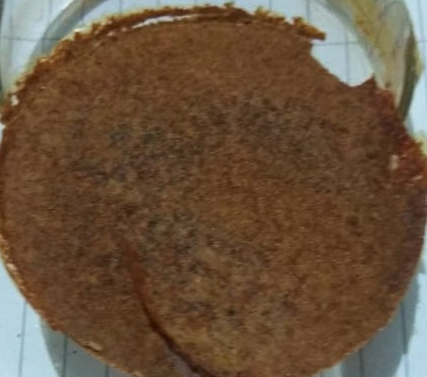OFG-*cl*-CH/catechin nanocomposite hydrogel**  **(d)** |

**Fig.S2 (a-d): (a-b) Pictorial representation of (a,b) OFG*-cl*-CH Schiff’s base hydrogel (c) catechin extracted from *Acacia catechu* heartwood (d) OFG-*cl*-CH/catechin nanocomposite hydrogel.**

**Fig.S3: Schematic representation of formation of OFG and OFG*-cl*-CH-catechin nanocomposite**

**Fig.S4: Schematic representation of MTX loading in OFG-*cl*-CH-catechin nanocomposite**


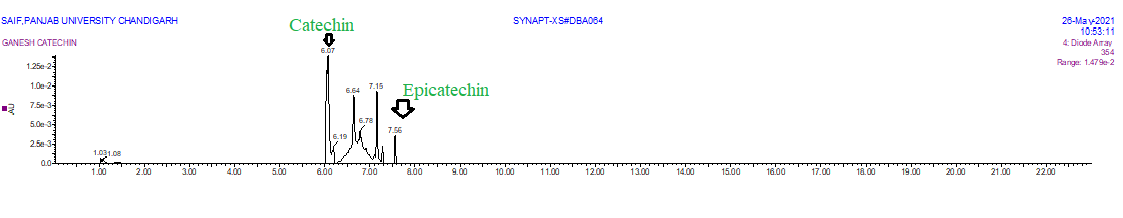


**Fig.S5: Total ion chromatograms of catechin extracted from *Acacia catechu*.**


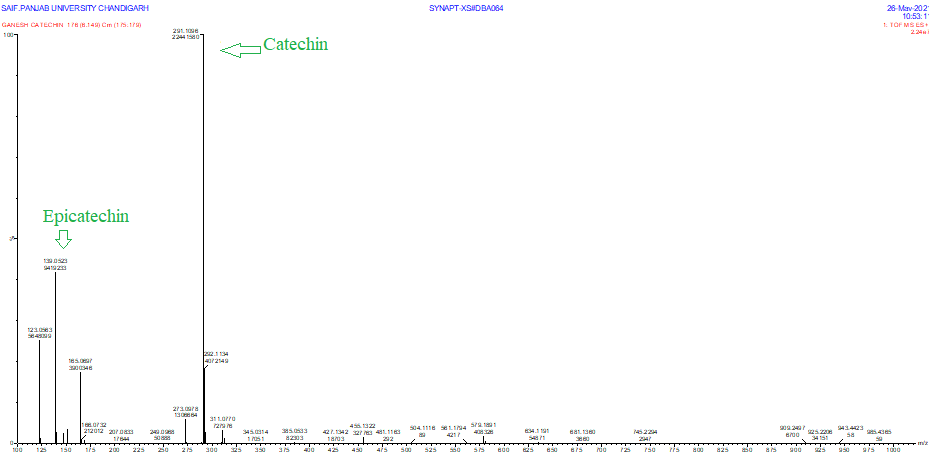


**Fig.S6: LC/MS spectrum of catechin extracted from *Acacia catechu*.**

**(a)
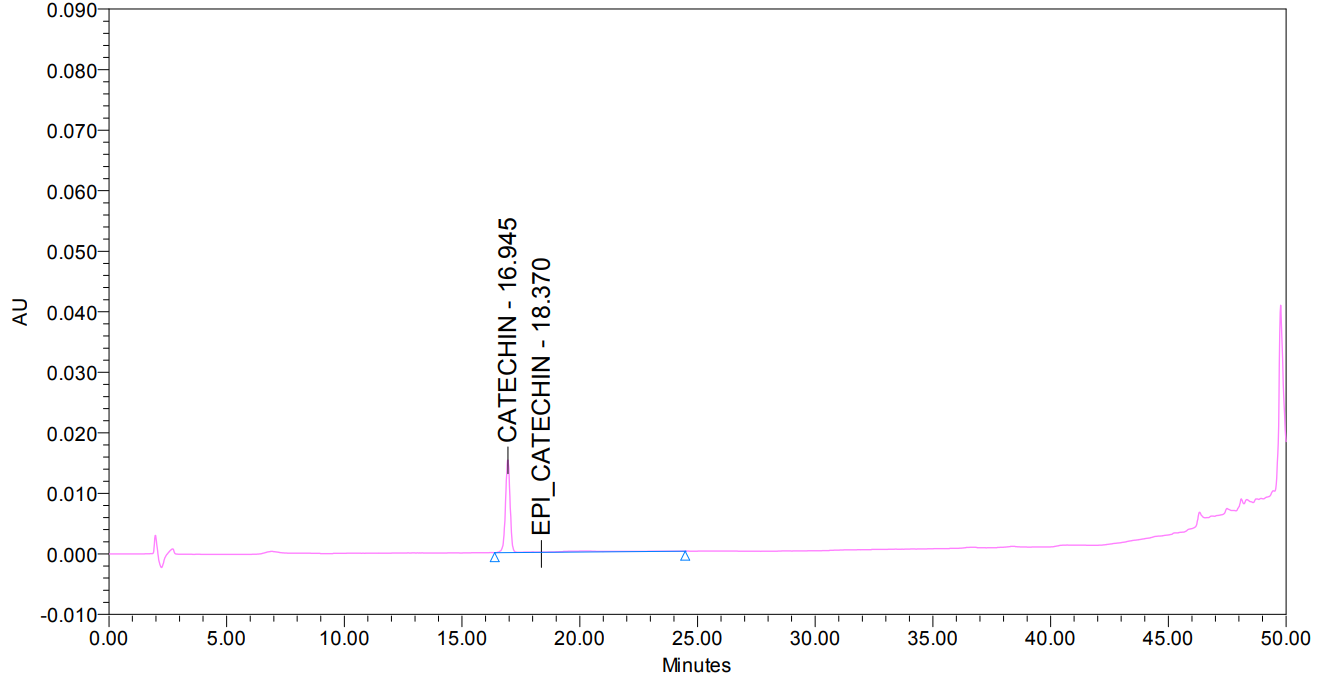
**

**(b)
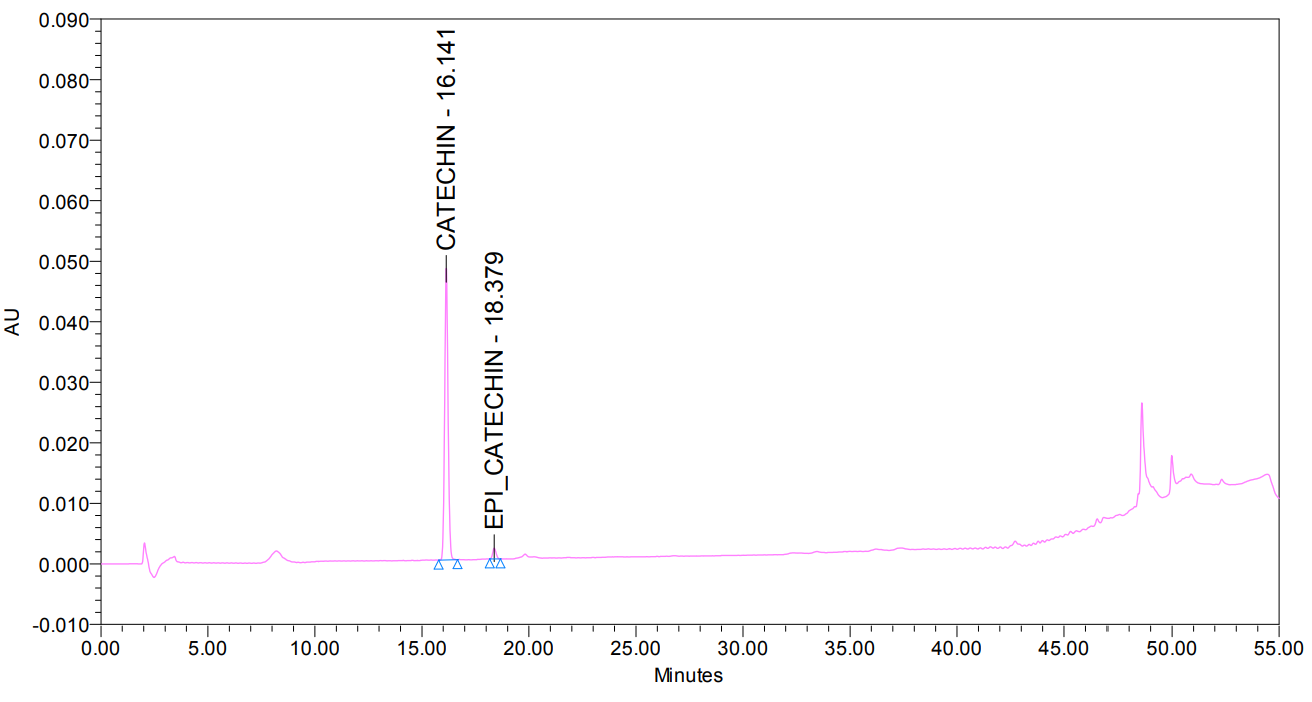
**

**Fig.S7: (a) HPLC chromatogram of standard catechin (600 µg/ml) at 280 nm; (b) HPLC chromatograms of catechin (600 µg/ml) extracted from *Acacia catechu*at 280nm.**

|   (a) | (b) |
| --- | --- |
| (c) | (d) |
| **Fig.S8 (a-d): (a) Swelling profile of OFCC nanocomposite gel matrix (catechin 5000µg/ml, encapsulated by *insitu* method);(b)Plot of *ln*W_t_/W_∞_ versus *ln t*; (c) Plot of W_t_/W_∞_ versus √ t; (d) Plot of *ln*(1-W_t_/W_∞_) versus Time, for OFCC nanocomposite gel matrix as a function of pH.** | |

|  |  |
| --- | --- |
|  |  |
| **Fig.S9(a-d):(a) Swelling profile of OFCC nanocomposite (catechin 2500µg/ml, encapsulated by swelling equilibrium method);(b)Plot of *ln*W_t_/W_∞_ versus *ln* t;(c)Plot of W_t_/W_∞_ versus √ t; (d) Plot of *ln*(1-W_t_/W_∞_) with Time, for OFCC nanocomposite gel matrix as a function of pH.** | |

**Fig.S10(a-b):(a)*In vitro* release dynamics of MTX from drug loaded OFC-MTXgel matrix in different release mediums at 37^o^C(b)Cumulative (%) of total drug release from drug loaded OFC-MTX gel matrix in different release mediums at 37^o^C.**

**Fig.S11(a-d):(a) Plot of zero order (b)Plot of First order (c)Plot of Higuchi model (d) Plot of Korsmeyer-Peppas model for *in vitro* release dynamics of MTX from drug loaded OFC-MTX gel matrix in different release mediums at 37^o^C.**

**Fig.S12(a-b): (a) Plot of M_t_/M_∞_versus t^1/2^ to calculate initial and average diffusion coefficient (D_i_& D_A_)and (b)Plot of *ln*(1-M_t_ /M_∞_) versus Time(min.) to calculate late diffusion coefficient (D_L_)for MTX release fromdrug loaded OFC-MTX gel matrix in different release mediums at 37^o^C.**

**
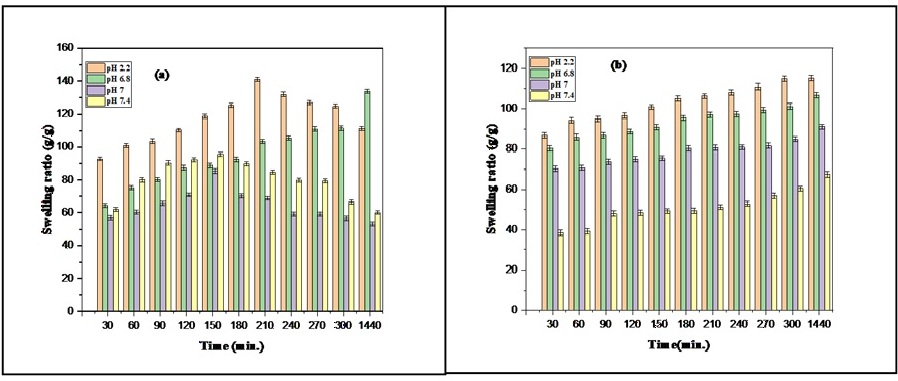
**

**Fig.S13(a-b): Swelling-shrinking phenomenon of (a) OFG-*cl*-CH Schiff’s base hydrogel (Fenugreek gum 2 %(w/v); Chitosan 4 % (w/v); NaIO_4_ 0.05M);(OFC),(b) OFG-*cl*-CH-catechin nanocomposite hydrogel (catechin 2500µg/ml, encapsulated by *insitu* method);(OFCC).**

| **Table-S1(a):Composition of OFG-*cl*-CH (OFC),OFG-*cl*-CH-catechin (OFCC),OFG-*cl*-CH-MTX (OFC-MTX) and OFG-*cl*-CH-catechin-MTX(OFCC-MTX) hydrogel formulations.** | | | | | | | |
| --- | --- | --- | --- | --- | --- | --- | --- |
| **Hydrogel**  **Matrix** | **OFG**  **(%)** | **CH**  **(%)** | **Catechin**  **(µg/ml)** | **MTX**  **(µg/ml)** | **Solvent** | **Initiator/**  **catalyst** | **Gelation**  **condition** |
| OFC | **2** | **4** | **-** | **-** | Double Distilled Water (DDW) | No | Room Temperature |
| OFCC | **2** | **4** | **2500** | **-** |  |  |  |
| OFC-MTX | **2** | **4** | **-** | **300** |  |  |  |
| OFCC-MTX | **2** | **4** | **2500** | **300** |  |  |  |

| **Table-S1(b): Qualitative phytochemical analysis of catechin extracted from *Acacia catechu* heartwood. (+ =Present, - = Absent)** | | |
| --- | --- | --- |
| **S.No.** | **Qualitative tests**  **(Phytoconstituents)** | **Catechin (extracted from *Acacia catechu*)** |
| 1. | Phenols | + |
| 2. | Alkaloids | + |
| 3. | Tannins | - |
| 4. | Steroids | + |
| 5. | Flavonoids | + |
| 6. | Carbohydrates | + |
| 7. | Glycosides | - |
| 8. | Saponins | - |
| 9. | Terpenoids | + |
| 10. | Sugar | - |

| **Table-S1(c): HPLC analysis of Standard Catechin and Catechin extracted from *Acacia catechu* heartwood at 280nm** | | | | | | | | | | | | |  |
| --- | --- | --- | --- | --- | --- | --- | --- | --- | --- | --- | --- | --- | --- |
|  |  | **Standard Catechin** | | | |  |  | | **Catechin extracted from *Acacia catechu* heartwood** | | | | |
| **S.No.** | **Peak Name** | **Retention Time(Minutes)** | **Area** | **%Area** | **Height** |  |  | **Retention Time(Minutes)** | | **Area** | **%Area** | **Height** |  |
| 1. | **Catechin** | 16.945 | 230489 | 100.00 | 15309 |  |  | 16.141 | | 531058 | 96.50 | 48403 |  |
| 2. | **Epi-catechin** | 18.370 | - | - | - |  |  | 18.379 | | 19246 | 3.50 | 1773 |  |

| **Table: S2: TGA analysis of FG, OFG, OFC gel matrix and OFCC nanocomposite gel matrix.** | | | | | | | | | | |
| --- | --- | --- | --- | --- | --- | --- | --- | --- | --- | --- |
| **Decomposition temperature (^o^C) for every 10% weight loss** | | | | | | | | | | |
| **Sample** | **IDT**  **(^o^C)** | **FDT**  **(^o^C)** | **10** | **20** | **30** | **40** | **50** | **60** | **70** |  |
| FG | 146.85 | 507.25 | 58.60 | 103.41 | 277.52 | 297.71 | 307.13 | 313.18 | 319.24 |  |
| OFG | 134.43 | 558.80 | 125.70 | 205.36 | 270.68 | 292.73 | 306.28 | 313.07 | 317.81 |  |
| OFC | 110.46 | 505.91 | 60.79 | 210.15 | 253.59 | 294.57 | 312.78 | 322.31 | 337.88 |  |
| OFCC | 150.90 | 575.73 | 55.12 | 125.85 | 224.11 | 272.12 | 305.26 | 317.73 | 330.55 |  |
